# Genome‐wide diversity and habitat underlie fine‐scale phenotypic differentiation in the rainbow darter (*Etheostoma caeruleum*)

**DOI:** 10.1111/eva.13135

**Published:** 2020-10-07

**Authors:** Daniel R. Oliveira, Brendan N. Reid, Sarah W. Fitzpatrick

**Affiliations:** ^1^ Department of Biology Clark University Worcester MA USA; ^2^ W.K. Kellogg Biological Station Michigan State University Hickory Corners MI USA; ^3^ Department of Integrative Biology Michigan State University East Lansing MI USA; ^4^ Ecology, Evolution, and Behavior Program Michigan State University East Lansing MI USA

**Keywords:** adaptive potential, *Etheostomacaeruleum*, genetic diversity, morphology, RADseq, thermal tolerance

## Abstract

Adaptation to environmental change requires that populations harbor the necessary genetic variation to respond to selection. However, dispersal‐limited species with fragmented populations and reduced genetic diversity may lack this variation and are at an increased risk of local extinction. In freshwater fish species, environmental change in the form of increased stream temperatures places many cold‐water species at‐risk. We present a study of rainbow darters (*Etheostoma caeruleum*) in which we evaluated the importance of genetic variation on adaptive potential and determined responses to extreme thermal stress. We compared fine‐scale patterns of morphological and thermal tolerance differentiation across eight sites, including a unique lake habitat. We also inferred contemporary population structure using genomic data and characterized the relationship between individual genetic diversity and stress tolerance. We found site‐specific variation in thermal tolerance that generally matched local conditions and morphological differences associated with lake‐stream divergence. We detected patterns of population structure on a highly local spatial scale that could not be explained by isolation by distance or stream connectivity. Finally, we showed that individual thermal tolerance was positively correlated with genetic variation, suggesting that sites with increased genetic diversity may be better at tolerating novel stress. Our results highlight the importance of considering intraspecific variation in understanding population vulnerability and stress response.

## 1 INTRODUCTION

Contemporary extinctions surpass background rates by orders of magnitude, signaling an unprecedented global defaunation period (Dirzo et al., 2014; Young et al., 2016). Despite rapid and steep declines in biodiversity, many natural populations can persist through environmental change, whether it be through ecological mechanisms, evolution, or both (DeLong et al., 2016; Kinnison & Hairston, 2007; Lande & Shannon, 1996; Willi & Hoffmann, 2009). In the face of global change, there are three principal modes of population response to avoid extinction (Bell & Collins, 2008; Gienapp et al., 2008; Norberg et al., 2012). First, organisms can disperse to more suitable environments in other locations (Chen et al., 2011; Parmesan et al., 1999). Second, they can remain and alter their responses through phenotypic plasticity, without changing their genetic composition (Charmantier et al., 2008; Nussey et al., 2005). Third, populations can evolve novel adaptations to stressful conditions through evolutionary rescue, enabling persistence despite environmental change (Kitano et al., 2008; Phillips & Shine, 2006).

In dispersal‐limited species, understanding the potential for plasticity and/or evolutionary rescue in facilitating population persistence is crucial (Reed et al., 2011). Plastic responses are capable of buffering against extinction within a range of environmental conditions when migration to more suitable conditions is not possible (Ghalambor et al., 2007; Reed et al., 2010; Sultan & Spencer, 2002). Similarly, when populations are dispersal‐limited and lack phenotypic plasticity, evolutionary rescue, or adaptive change that prevents extinction due to maladaptation to novel conditions, can reduce population decline in the face of environmental change (Gomulkiewicz & Holt, 1995; Gonzalez et al., 2013; Thompson, 1998). The potential for evolutionary rescue relies largely on whether a population contains the necessary standing genetic variation to respond to selection (Barrett & Schluter, 2008; Gibson & Dworkin, 2004; Paaby & Rockman, 2014). Thus, one concern for many species is that they may lack the genetic variation necessary to adapt, thereby facing an increased extinction risk (Bohonak, 1999; Massot et al., 2008; Thomas et al., 2004).

Beyond innate dispersal capability, barriers to connectivity can also have a significant impact on population persistence. For instance, reduced connectivity due to habitat fragmentation or degradation may lead to higher genetic drift, which in turn reduces genetic diversity and increases the likelihood of local extinction (Dixo et al., 2009; Fahrig, 2003; Wilcox & Murphy, 1985). In many freshwater systems, connectivity relies on the hierarchical and dendritic topology of streams (Paz‐Vinas & Blanchet, 2015; Carrara et al., 2014). Spatial and environmental heterogeneity in these systems facilitates local adaptation and biological diversification (Carrara et al., 2012). However, freshwater environments suffer disproportionately from habitat fragmentation compared with other landscape networks due to their unique hierarchical structure (Fagan, 2002), often resulting in reduced connectivity and demographic decline. Small and isolated populations, such as those found in headwaters or blocked stream sections, are vulnerable to genetic threats such as reduced adaptive potential and the deleterious effects of inbreeding (Gaggiotti, 2003; Willi et al., 2006). This is often illustrated in the heterozygosity–fitness relationship, by which populations with decreased heterozygosity (reduced genetic diversity) likewise suffer from a reduction in fitness or stress tolerance (Frankham, 1996, 2005; Reed & Frankham, 2003). The combined environmental, demographic, and genetic threats in isolated populations, especially those restricted to isolated stream sections, can result in a rapid decline in population size and eventual extinction, a process known as the extinction vortex (Blomqvist et al., 2010; Fagan & Holmes, 2006).

Freshwater fishes have the highest extinction rate among vertebrates in the twentieth century, largely attributed to habitat loss and modification (Mantyka‐Pringle et al., 2014; Burkhead, 2012). In addition, climate change and increased temperatures pose other significant yet understudied threats (Comte et al., 2013; Daufresne & Boët, 2007). Cold‐water habitats in the United States are predicted to decrease by nearly 50% given a simulated doubling of atmospheric carbon dioxide to 660 ppm (Boer et al., 1992; Eaton & Scheller, 1996), leading to a substantial projected decline of cold‐water species (Comte et al., 2013). For species restricted to cold, headwater habitats, the capacity for movement to a more suitable location is likely limited. Therefore, characterizing contemporary variation in thermal tolerance and the potential to “adapt in place” is critical for determining the potential for population persistence given associated increases in stream temperatures.

North American darters in the family Percidae are a speciose clade (>200 species) consisting of mostly small fish that occupy fast‐flowing and generally cool‐water streams (Kuehne & Barbour, 1983; MacGuigan & Near, 2019; Near et al., 2011). Many species are sympatric, exhibiting reproductive isolation through sexually dimorphic characteristics (such as nuptial coloration) and assortative mating (Mendelson, 2003; Mendelson et al., 2007). Although the phylogenetic history of this clade has been well‐studied, intraspecific eco‐evolutionary dynamics, contemporary population structure, and population‐level persistence have received less attention. Many darter species are microendemic to single streams, rendering them vulnerable to extinction (Meyer et al., 2007). In fact, 88 darter species are listed as threatened, vulnerable, or endangered, with the Maryland darter likely extinct (Jelks et al., 2008). Amidst these widespread declines, there is a lack of fundamental knowledge on the adaptive potential of darters and their responses to novel stressors such as temperature.

In this study, we use a common species of darter to address questions about intraspecific variation and vulnerability, providing insight for more threatened species. The rainbow darter (*Etheostoma caeruleum*) is a common darter species found throughout the Eastern and Central United States, primarily in the Upper Mississippi River Basin and Great Lake drainages. Although rainbow darters have a widespread distribution, studies of other darter species have shown that populations potentially suffer from decreased connectivity and reductions in habitat quality (Fitzpatrick et al., 2014; Fluker et al., 2010). Previous population genetic studies on the rainbow darter based on mitochondrial and microsatellite data found haplotype differentiation throughout the range (Ray et al., 2006), significant genetic differentiation between populations from the Lake Erie and Ohio River catchments (Haponski et al., 2009), and genetically diverse populations with relatively little structure across Iowa (Davis et al., 2015). However, the minimum spatial scale at which rainbow darters exhibit adaptive differentiation, and how genetic variation affects stress response, is not known.

The specific aims of this study were to first, characterize patterns of phenotypic and genetic variation in the rainbow darter on a local scale, and second, test what factors affect thermal stress tolerance. Whereas this species is typically restricted to stream environments, our study also included a unique site from a large glacial lake, only the second‐ever documented lake occurrence of rainbow darters (Katula, 2013). We were interested in testing the hypothesis that adaptive potential varies among sites, evidenced by phenotypic and genetic differentiation associated with local environmental conditions. We addressed the following questions: (a) Do rainbow darters exhibit phenotypic differentiation associated with lake versus stream environments? (b) What are the patterns of within and among population genetic variation on a local scale? and (c) Does the environment and/or individual heterozygosity affect thermal tolerance? Our study contributes to understanding the scale of population differentiation in a common darter species, and the potential sensitivity of this cold‐water species to environmental change.

## 2 METHODS

### 2.1 Study area and sampling

**Figure 1:**
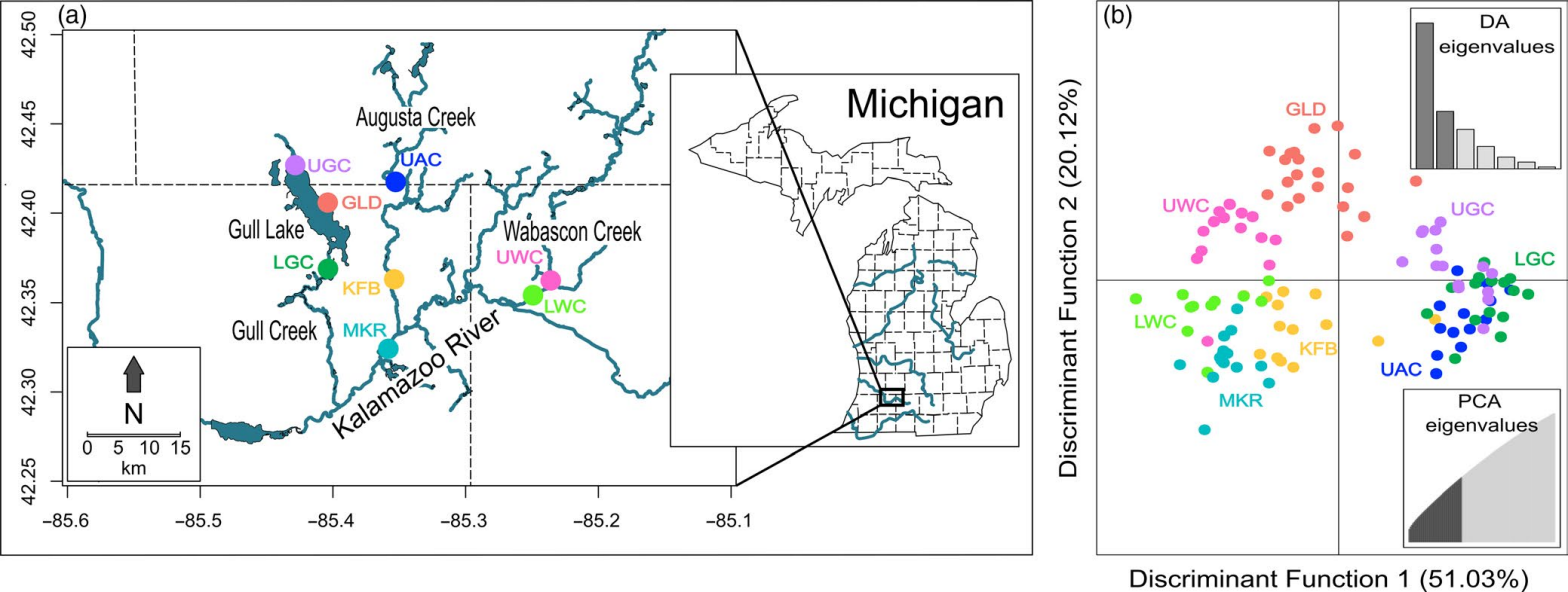
(a) Map showing eight sample sites across three tributaries (Gull, Augusta, and Wabascon Creek) of the Kalamazoo River. (b) Population structure results from the discriminant analysis of principal components (DAPC), with discriminant functions one and two represented on the x and y axes, respectively. Each point corresponds to an individual and colors reflect each sampling site. The top right inset displays eigenvalues for each discriminant function and the bottom right inset indicates the number of principal components retained in the analysis (43)

Sampling was conducted throughout June–August 2017 and 2018, across eight sample sites in Kalamazoo, Barry, and Calhoun Counties in Southwestern Michigan, USA (Figure 1a; Table 1). We chose paired upstream and downstream sites from three tributaries of the Kalamazoo River (Augusta Creek, Gull Creek, and Wabascon Creek). In addition, we included one mainstem site on the Kalamazoo River and one lake site (Gull Lake) that was situated between the paired stream sites in Gull Creek.

**Table 1:** Sample collection data

Sample site	Tributary	Year sampled	Habitat type	Number of individuals phenotyped	Number of individuals genotyped	Latitude	Longitude
Kellogg Forest (KFB)	Augusta	2017–2018	Stream	23	15	42.3633°	−85.3533°
Upper Augusta Creek (UAC)	Augusta	2017–2018	Stream	24	15	42.4176°	−85.3524°
Lower Gull Creek (LGC)	Gull	2017	Stream	19	15	42.3686°	−85.4037°
Upper Gull Creek (UGC)	Gull	2017	Stream	19	15	42.4268°	−85.4281°
Gull Lake (GLD)	Gull	2017–2018	Lake	25	24	42.4063°	−85.4041°
Upper Wabascon Creek (UWC)	Wabascon	2018	Stream	15	15	42.3623°	−85.2359°
Lower Wabascon Creek (LWC)	Wabascon	2018	Stream	12	12	42.3541°	−85.2492°
Kalamazoo River (MKR)	Kalamazoo	2018	Stream	16	16	42.3241°	−85.3584°

Note

Sample site and tributary correspond to Figure 1a, habitat type refers to either a lake or stream environment, number of individuals phenotyped refers to the number of fish per site included in morphological analyses, number of individuals genotyped refers to the number of fish per site included in the RADseq analyses.

We collected rainbow darters (*N* = 12–26 per site) from each site using a combination of seine nets and hand nets for a total of 156 individuals. We included both males and females in our sampling, but avoided sampling juveniles, defined as individuals with standard length < 25mm. During sampling, individuals were held streamside in plastic buckets filled with site water until completion of sampling. We transported individuals from each site in Nalgene^®^ containers filled with site water to the Experimental Pond Lab at W.K. Kellogg Biological Station in Hickory Corners, Michigan. We collected water temperature data from each site using a YSI Pro20 (YSI Inc.) on the date of sample collection. Additionally, we obtained the estimated average maximum air temperature for each site from June–August using daily values from the Daymet dataset, which extrapolates temperatures at a 1 km^2^ scale using weather station data (Thornton et al., 2018).

Of the 156 individuals, 25 were collected from three sites that we sampled in 2017 (eight individuals from Kellogg Forest and Upper Augusta Creek each, and nine from Gull Lake) and then were resampled in 2018 to account for potential temporal variability in phenotypes. The only individuals from resampled sites in 2018 that were included in the genomic data collection were those from Gull Lake, to increase sample size at this site.

### 2.2 Phenotypic measurements

Immediately upon arrival to the laboratory, each fish was anesthetized in a dilute solution of tricaine mesylate (MS‐222), weighed, measured (standard length), and photographed. We took photographs under fluorescent lighting of each individual spread laterally behind a measured background in water using a Canon EOS Rebel T3 digital camera (Canon U.S.A. Inc.). Rainbow darters from the same site were housed in 20‐gallon group tanks with a maximum of 10 individuals per tank. We prepared these tanks identically, including filters, water circulation devices, aquarium gravel, and bricks and plastic plants for refugia. Water was dechlorinated, denitrified, treated for ammonia and heavy metals, and boosted with aquatic microbiota (Ecological Laboratories Inc.) before use in tanks. We fed fish daily, alternating between frozen brine shrimp and bloodworms with amounts based on the number of individuals per tank. The room was lit with natural light from windows, following daily light/dark cycles. All fish were acclimated to a similar ambient water temperature of 24.5 ± 3°C.

After a minimum of 24 hr in the laboratory, we measured and defined individual thermal tolerance using the critical thermal maximum (CT_max_). We defined CT_max_ as the temperature threshold at which loss of equilibrium or organized locomotion occurs, signaling individuals have reached their physiological limit prior to death (Becker & Genoway, 1979). Specifically, this limit is characterized by rapid muscle and operculum spasms followed by keeling or a loss of an ability to remain an upright position. For ectotherms, the rate of temperature increase can have different biological implications depending on the size of the organism, particularly for fish species (Lutterschmidt, 1997). Due to the small size of rainbow darters, we chose a low rate relative to other studies to limit lag between temperature increase and physiological response. The rate of temperature increase was chosen as 0.33°C per minute and was calculated at a set transformer voltage of either 100 or 120 V depending upon the immersion heater used.

Prior to trials, individuals were starved for 24 hr. An insulated, rectangular (48 cm × 34 cm × 18 cm) Coleman^®^ 31‐quart cooler was filled with a constant volume (three gallons) of room temperature, dechlorinated, and denitrified water treated for ammonia and heavy metals. Two aeration devices were used to prevent a decline in dissolved oxygen content due to increasing temperatures. A 2.8 watt Rio^®^ Plus 90 Aqua Pump (TAAM Inc.) generated a current to maintain temperature equilibrium throughout the cooler, while two immersion heaters connected to a voltage transformer (PHC Enterprise Inc.) were placed on opposite ends of the cooler to facilitate gradual temperature increase. Reusable coffee filter containers covered with mesh netting were used to contain individuals during trials. Individuals were acclimated in the water for 10 min before beginning trials. We tested one fish per container, with a maximum of four individuals tested per trial. During trials, temperature increase was recorded using a YSI Pro20, and individuals were monitored for visual indications of reaching CT_max_. Upon reaching CT_max_, individuals were removed from the trial and given a recovery period to ensure survival. A single fish in our study failed to recover, likely due to surpassing its CT_max_. This individual was not included in subsequent CT_max_ analyses. After each CT_max_ trial, we used sterile scissors to clip one anal fin from all recovered individuals. Tissues were stored within 1.5 ml tubes containing 100% ethanol for subsequent genomic analyses. After recovery was confirmed, we euthanized individuals using a lethal dose of buffered MS‐222.

### 2.3 Morphometric analyses

**Figure 2:**
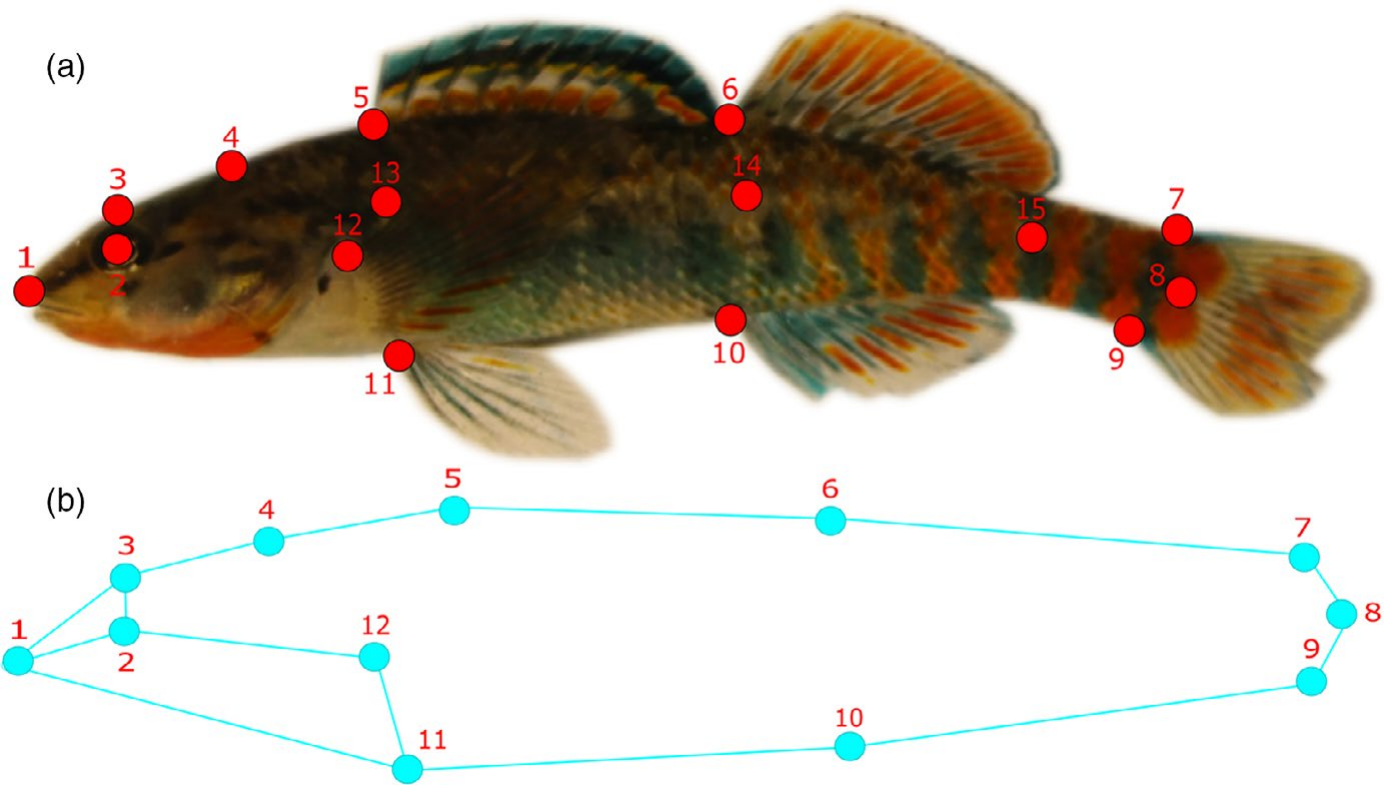
Fifteen landmarks used for the geometric morphometric analysis: (1) Anterior point on the head, (2) center of the eye, (3) landmark directly above the eye, (4) point at the supraoccipital notch, anterior junction of the (5) first and (6) second dorsal fins with the dorsal midline, (7) dorsal junction of caudal tail, (8) junction of lateral line and caudal tail, (9) ventral junction of the caudal tail, (10) anterior junction of the anal fin with the ventral midline, (11) anterior junction of the pelvic fin with the body, (12) dorsal junction of the pectoral fin with the body, (13–15) landmarks along the lateral line accounting for organismal bending. (a) Landmarks placed on an actual rainbow darter. (b) Wireframe graphic of landmarks produced in *MorphoJ*. Landmarks used to correct for bending (13–15) are not included

To test for differences in morphology among sites and habitat, we conducted geometric morphometric analyses. We compiled JPEG images from 154 individuals into a TPS file using *tpsUtil v1.76* (Rohlf, 2018). Each image was scaled and digitized with 15 anatomical landmarks in *tpsDig v2.30* (Rohlf, 2017). We used a series of modified landmarks (Guill et al., 2003), with three landmarks serving to correct bend curvature bias (Figure 2). Landmarks used for correcting curvature in organisms were removed prior to downstream analysis due to not corresponding to actual homologous regions across individuals. Bend curvature was corrected using *tpsUtil* and subsequent analysis of body shape variation was carried out using the *MorphoJ* software (Klingenberg, 2011). Individuals were superimposed using a Procrustes fit to obtain Procrustes coordinates that accounted for variation associated with size and position of landmarks for each individual (Adams et al., 2004). Subsequently, we carried out a Procrustes ANOVA to evaluate support for site differences in overall body shape. We included site as a fixed effect and sex as a covariate. A canonical variate analysis (CVA) was then employed to identify the main axes of variation in body shape and calculate significant pairwise differences in shape among sites. This type of analysis is useful when seeking to explore shape variation for multiple groups with known a priori grouping. A permutation test for significance was conducted on pairwise Procrustes distances between sites with 10,000 iterations. To assess morphological divergence between habitat types, we conducted a discriminant function analysis (DFA) between the two habitats (stream (*N* = 130) versus lake (*N* = 24)). In order to determine the effect of sex, we also carried out a DFA between males (*N* = 70) and females (*N* = 84) for all sites. For each DFA, a permutation test with 10,000 iterations was used to assess significance between groups, while a cross‐validation analysis determined likely individual group membership. Wireframe graphs were used for visualizing the direction of morphological change associated with each canonical variate and discriminant function axis.

### 2.4 Molecular methods

Total genomic DNA was extracted from fin clips using a DNeasy Blood and Tissue Kit, according to manufacturer protocols (QIAGEN). After extraction, individual DNA concentration was quantified using either a Qubit 3.0 fluorimeter dsDNA HS Assay kit (Thermo Fisher Scientific) or by Quant‐iT™ PicoGreen™ assay (Thermo Fisher Scientific). Library preparation followed a modified restriction site‐associated DNA sequencing (RADseq) protocol described by Ali et al. (2016). Briefly, samples were divided into two libraries, with individuals concentrated to a target DNA of 120 ng in 10 µl total volume. Samples were digested using the SbfI restriction enzyme and ligated with a unique, eight base pair length adapter sequence unique to each library. Samples were pooled by library and then sonicated to ~ 500 base pair fragments using a M220 Focused‐ultrasonicator™ (Covaris). Sonicated DNA was ligated to Dynabead^®^ M‐280 Streptavidin beads for a series of four washes, after which the DNA was liberated using the SbfI restriction enzyme. Preparation for sequencing followed the protocol outlined in the NEBNext Ultra DNA Library Prep Kit for Illumina with no modifications (New England Biolabs). Six base pair length adapter sequences were added to identify replicate individual adapter sequences between the two libraries. Agencourt^®^ AMPure XP (Beckman Coulter) bead size selection was performed based on the DNA concentration obtained from a Qubit 3.0 fluorimeter. The final libraries were submitted to the RTSF Genomics Core at Michigan State University and were pooled for sequencing in one lane with paired‐end 150 base pair reads on an Illumina HiSeq 4000.

### 2.5 RADseq analyses

Forward and reverse sequence data were reoriented and reads without barcodes were removed using *Flip2BeRad* (https://github.com/tylerhether/Flip2BeRAD). We then used *stacks v2.0* (Catchen et al., 2011, 2013; Rochette et al., 2019) to filter, trim adapter sequences, demultiplex, and call genotypes (Table S2). First, the *clone_filter* function was used to identify and remove PCR clones. We then ran the *process_radtags* function to trim poor‐quality adapter sequences and restriction site matches, allowing for a two base pair mismatch in rescuing barcode sequences. Reads were then demultiplexed and low‐quality reads removed (Table S3). Forward and reverse reads were aligned to a draft of the orangethroat darter genome (Moran et al., 2019) using the default settings of *BWA‐MEM* (Li, 2013) and a custom script (https://github.com/ryanpeek/radseq). Combining both libraries, the *ref_map.pl* function in *stacks* was used to identify single nucleotide polymorphisms (SNPs), calling only the first SNP per RAD locus. We only kept SNPs that were found in all eight sites, were present in 75% of individuals within each site, and with a minimum minor allele frequency threshold of 0.05. We further filtered the data to remove individuals with more than 75% missing data, excluding eight individuals, using *VCFtools v0.1.15* (Danecek et al., 2011). Downstream analyses were conducted on file formats outputted from *stacks* or converted using *PGDSpider v2.1.1.5* (Lischer & Excoffier, 2012). In total, after filtering we identified 7,906 SNP loci.

### 2.6 Genetic diversity and population structure

The output from *populations* in the *ref_map.pl* function of *stacks* was used to analyze among site differentiation in observed and expected heterozygosity, nucleotide diversity, and pairwise F_ST_. Within site relatedness was calculated using identity by descent in *Plink v1.9* (Chang et al., 2015).

Among site differentiation was determined using discriminant analysis of principal components (DAPC) in the R package *adegenet* (Jombart, 2008; Jombart & Ahmed, 2011; R Core Team, 2018). This method is useful for discovering patterns of genetic clustering, with the option of grouping individuals a priori. Since we were interested in maximizing the differentiation between sample sites rather than genetic clusters, we chose to use a cluster value of eight, assigning individuals according to sample site. To find the number of informative principal components to retain, we carried out a cross‐validation analysis with 1,000 repetitions. Patterns of admixture were assessed using *ADMIXTURE v1.3* (Alexander et al., 2009). The number of genetic clusters best fitting the data was calculated by performing a 10‐fold cross‐validation in *ADMIXTURE*, resulting in the highest support for two genetic clusters (*k* = 2). We carried out ten independent runs with two genetic clusters (*k* = 2). Results from these ten runs were visualized using the *pophelper* package in R (Francis, 2017) and consensus ancestry between the ten runs was calculated using *CLUMPP* (Jakobsson & Rosenberg, 2007) in *pophelper*.

In order to determine the effects of geography on genetic diversity and potential migration, we tested for signatures of isolation by distance (IBD). Geographic distances between sites were collected by following stream networks on Google Earth (Table S4). Pairwise F_ST_ between sites, calculated from *stacks*, was used as a metric of genetic distance. A Mantel test was carried out in the *ade4* package in R (Dray & Dufour, 2007) between geographic and genetic distance, with 10,000 permutations. To further test whether contemporary patterns of genetic differentiation are a product of stream connectivity between sites, we utilized *StreamTree* (Kalinowski et al., 2008). We assigned genetic distances for stream sections using pairwise F_ST_, testing whether observed site genetic differentiation reflects expected genetic distance based on stream connections. Therefore, it is possible to identify stream sections linked to higher than expected genetic differentiation, owing to reduced gene flow (due to known or unknown connectivity barriers). For both IBD and *StreamTree*, we visualized and tested for a significant correlation between distance metrics using the *JGR* package (Helbig et al. 2013) and a Spearman's rank correlation coefficient respectively in R.

### 2.7 Thermal tolerance and genetic diversity

We assessed whether genetic diversity influences CT_max_ by testing for a correlation between individual observed heterozygosity and CT_max_ using a Spearman's rank correlation coefficient. In order to determine whether local environmental temperature at a given sample site or observed heterozygosity better explained individual variation in CT_max_, we carried out a linear mixed‐effects model (Bates et al., 2015). We defined the full model as:

$${\mathrm{CT}}_{\max}=E+G+ExG+S+W+\left(1\right|C)+\left(1|B\right)+(1|Y)$$

where CT_max_ is the response variable for an individual. The fixed effects were defined as *E* (sample site), *G* (heterozygosity), *E x G* (interaction between site and heterozygosity), *S* (sex), and *W* (weight). We also included *C* (container position within cooler), *B* (trial number), and *Y* (year) as random effects. All models included weight and sex as fixed effects and container position, trial number, and year as random effects. In total, we tested five models: the null (neither site nor heterozygosity), only site (*E*), only heterozygosity (*G*), both site and heterozygosity (*E + G*), and both effects plus their interaction (*E + G + E x G*). We fit each model using maximum likelihood and used a likelihood ratio test to determine significance between models with different fixed effect terms. Plots of residuals were used to determine whether assumptions of normality and homoscedasticity were met for all models. The Akaike information criterion (AIC) was used in model selection to determine the model with the greatest explanatory power (Burnham & Anderson, 2004). Fine‐scale pairwise differences in CT_max_ among sites were further explored in the site only (*E*) model using the *emmeans* (Lenth et al., 2018) and *MuMIn* (Barton, 2018) packages in R. For resampled sites, we analyzed temporal variation in CT_max_ using a Student's *t* test. The correlation between site water temperature, average maximum air temperature, and CT_max_ was analyzed using a Spearman's rank correlation coefficient.

## 3 RESULTS

### 3.1 Morphological variation

**Figure 3:**
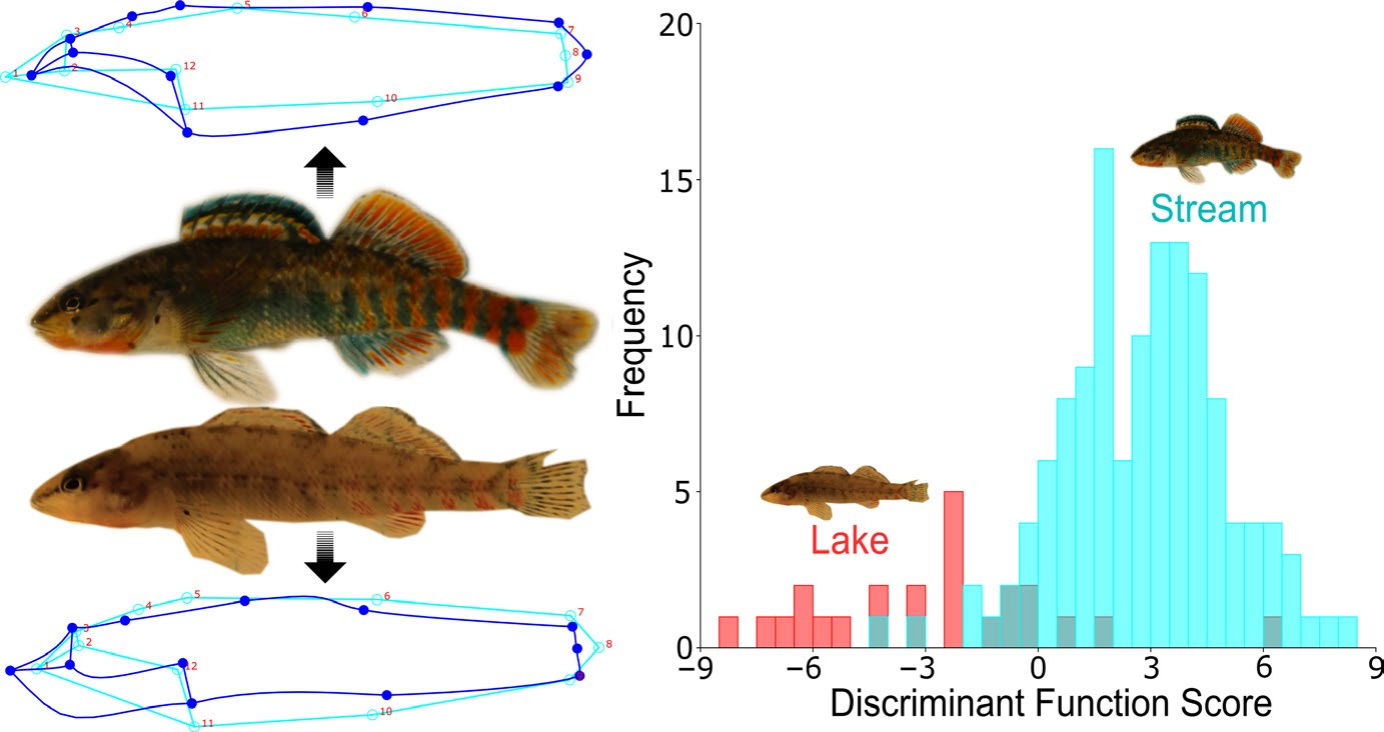
Scores produced from discriminant function analysis (DFA) conducted between the two different habitat types (lake versus stream). Stream individuals are represented by the images in the top left, while lake individuals are represented in the bottom left. Rainbow darter images are individuals from each extreme of the discriminant function, with the wireframe graphics magnified to highlight the direction of morphological differentiation

The DFA between habitats revealed significant differences in body shape between lake and stream individuals (Figure 3; *p* < .001). Body shape differences between the two groups are characterized by greater body depth and a terminal mouth position in stream individuals, while lake individuals display reduced body depth and a shift toward a superior mouth position. The cross‐validation analysis assigned eight lake individuals with the stream group (~33%), and 16 stream individuals with the lake group (~12%). We also found significant differences in body shape between sexes. Generally, sex differences were associated with subtle differences in body depth, with males having greater body depth. The cross‐validation analysis was less accurate, assigning 33 females as males (~39%) and 21 males as females (~30%). The Procrustes ANOVA for general differences in body shape found significant differences across sites (*F*(140, 2,900)=4.28, *p* < .001), and between sexes (*F*(20, 2,900)=3.19, *p* = .003). Despite significant differences indicated by the Procrustes ANOVA, the canonical variate analysis (CVA) between sites revealed a significant overlap in body shape among sites (Figure S1). The first two axes, canonical variate 1 (CV1) and canonical variate 2 (CV2), explained 69.83% of the total variance among sites. CV1, explaining 40.18% of the variance, was generally associated with posterior body depth and the pectoral fin. CV2, explaining 29.65% of the variance, reflected anterior differences in body depth and mouth position. Subsequent permutation tests revealed significant differences in body shape and morphological differences between many sites (Table S1). The two sites morphologically divergent from all others were Gull Lake (GLD) and Upper Augusta Creek (UAC).

### 3.2 Genetic diversity and population structure

Observed heterozygosity was highest in the mainstem Kalamazoo (MKR, 0.34) and Lower and Upper Wabascon Creek sites (LWC, 0.34 and UWC, 0.34), and lowest in Upper Augusta Creek (UAC, 0.30) and Lower Gull Creek (Table 2; LGC, 0.30). Nucleotide diversity was less consistent, with UAC and LGC having the lowest diversity for this metric and GLD and LWC the highest. Interestingly, GLD harbored higher genetic variation compared with its respective inlet and outlet. Relatedness also varied by site and was highest in UAC and LGC (0.19 and 0.17, respectively), above the threshold designating third‐degree relationships (Table 2). GLD and Kellogg Forest (KFB) had the lowest relatedness (0.02 each).

**Table 2:** Summary of genome‐wide diversity metrics for each sample site

Sample site	Observed heterozygosity (*SD*)	Expected heterozygosity (*SD*)	Nucleotide diversity (*SD*)	Relatedness (*SD*)
Kellogg Forest (KFB)	0.33 (0.20)	0.32 (0.15)	0.33 (0.15)	0.02 (0.03)
Upper Augusta Creek (UAC)	0.30 (0.22)	0.28 (0.17)	0.29 (0.18)	0.19 (0.05)
Lower Gull Creek (LGC)	0.30 (0.21)	0.28 (0.19)	0.29 (0.18)	0.17 (0.03)
Upper Gull Creek (UGC)	0.31 (0.20)	0.30 (0.16)	0.31 (0.16)	0.08 (0.05)
Gull Lake (GLD)	0.33 (0.18)	0.33 (0.14)	0.34 (0.15)	0.02 (0.03)
Upper Wabascon Creek (UWC)	0.34 (0.20)	0.32 (0.15)	0.33 (0.15)	0.03 (0.06)
Lower Wabascon Creek (LWC)	0.34 (0.21)	0.31 (0.15)	0.34 (0.16)	0.04 (0.13)
Kalamazoo River (MKR)	0.34 (0.20)	0.32 (0.15)	0.33 (0.15)	0.05 (0.06)

Note

*SD*, standard deviation.

**Figure 4:**
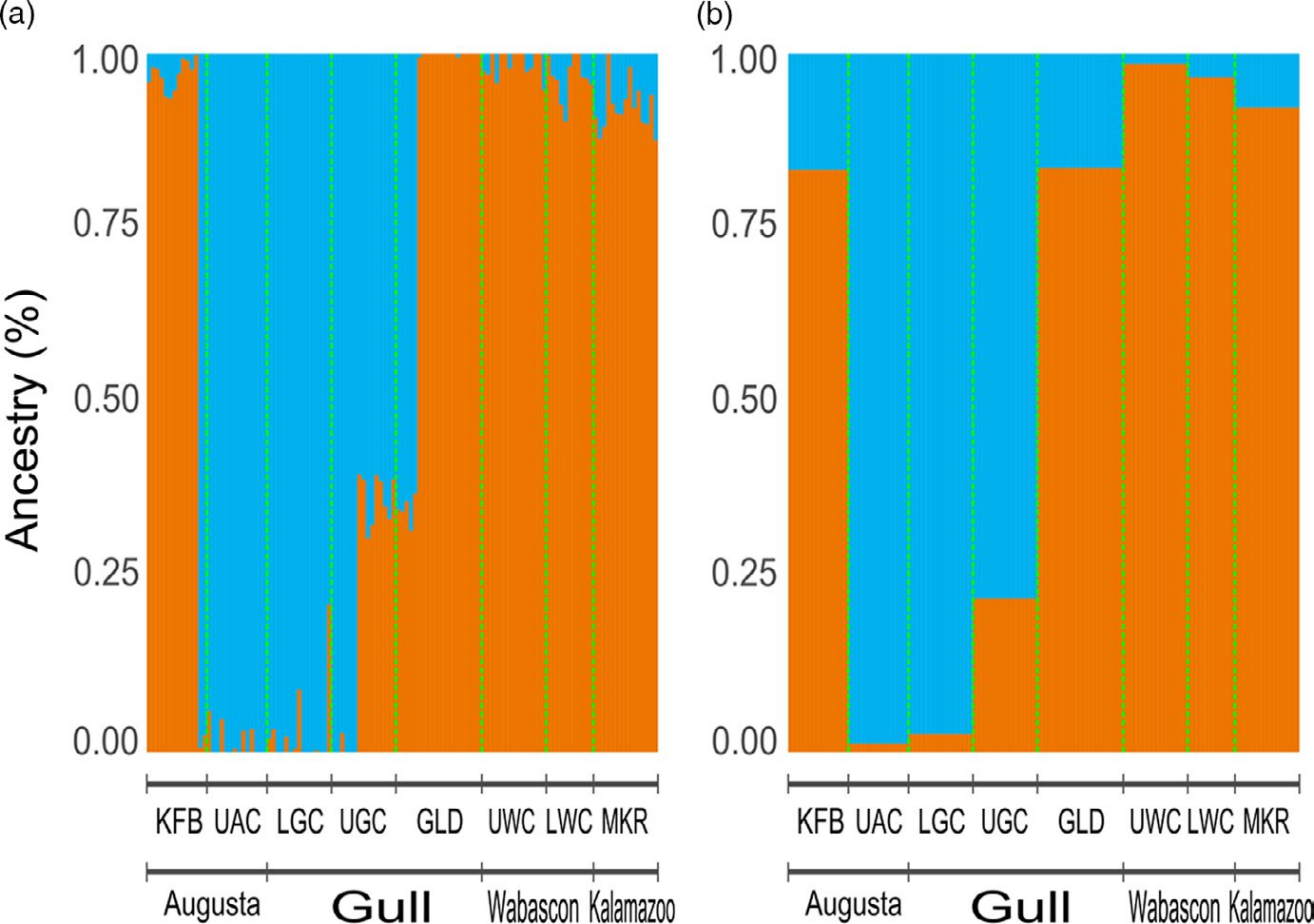
Population structure inferred from *ADMIXTURE* for (a) each individual and (b) the population mean for the two genetic clusters (*k* = 2). An individual is represented by a single vertical column, with the green dotted lines demarking sites

Pairwise genetic distance between sites (*F*_ST_) was generally low, ranging from 0.02 (between UAC and LGC) to 0.07 (Table S4; between UAC and LWC). Counterintuitively, *F*_ST_ between UAC and the paired downstream KFB site (0.06) was higher than the genetic distance between KFB and all other sites. The discriminant analysis of principal components (DAPC) was primarily defined by the first linear discriminant differentiating sites (Figure 1b). Although individuals within sites largely clustered together, Upper Gull Creek (UGC), UAC, and LGC grouped together, and one individual from KFB clustered with these sites. The first discriminant axis primarily differentiated individuals from Gull Creek and UAC from the Wabascon Creek and MKR sites, while the second discriminant axis revealed no clear population differentiation. Analyses from *ADMIXTURE* indicated similar population structure results, with *k* = 2 being the supported number of genetic clusters (Figure 4). Individuals from Gull Creek and UAC sites formed a unique cluster, while the remainder of the sites formed another cluster. Interestingly, there is evidence for admixture between the clusters in KFB, GLD, and UGC, with select individuals within these sites showing a mixed ancestry from both genetic clusters.

We found no evidence for isolation by distance (IBD), indicated by the nonsignificant correlation between genetic and geographic distance (Figure S2; *r*_s_ = 0.0328, *p* = .8684) and a nonsignificant Mantel test (*p* = .9911). Genetic divergence between sites was not explained by stream connectivity, with differentiation between sites being either greater or lower than expected based upon the genetic distance of each stream segment (Figure S3; *r*_s_ = 0.2637, *p* = .1752). Lack of support for the *StreamTree* model reinforced the absence of IBD, indicating a structuring of these sites not owing to stream connectivity (sites have either higher or lower observed genetic distance than is expected from cumulative stream genetic distance).

### 3.3 Thermal tolerance and genetic diversity

**Figure 5:**
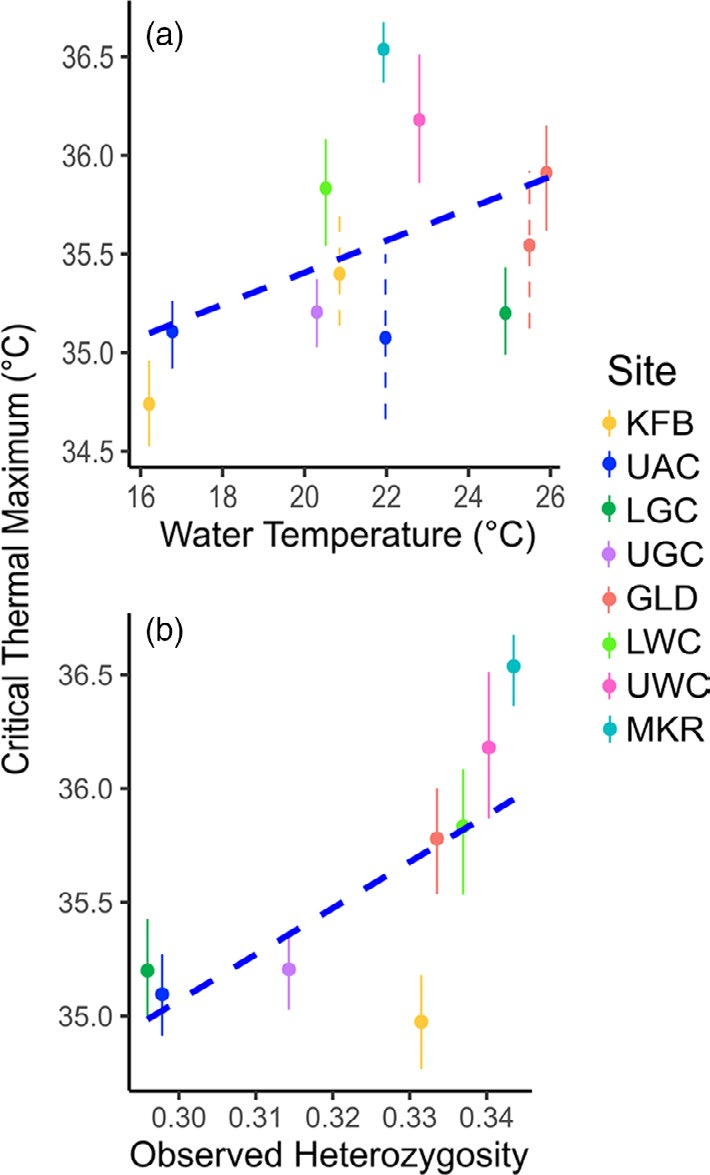
(a) Correlation between site water temperature and critical thermal maximum (CT_max_). Error bars indicate 95% confidence intervals with dashed error bars representing sites in which resampling was conducted in 2018. (b) Correlation between site observed heterozygosity from *stacks* and CT_max_. Error bars indicate 95% confidence intervals

Heterozygosity showed a significant correlation with CT_max_. For both observed (*r*_s_ = 0.2617, *p* = .004) and expected (*r*_s_ = 0.4402, *p* < .001) heterozygosity, we found a positive trend, in which sites with higher heterozygosity also had higher CT_max_ (Figure 5b). The top two supported models were the model including the additive effects for both site and observed heterozygosity (*E + G*) and the model including only site (*E*) (ΔAIC = 0.92, Χ^2^(1, *N* = 119)=2.92, *p* = .087); both models explained a significant proportion of variability in CT_max_ (Table S3; conditional *R* ^2^ = 0.7337 and 0.8144, respectively).

In the site only model (*E*), we found significant pairwise differences in critical thermal maximum (CT_max_), generally among tributaries, but also found within‐tributary differences in Gull Creek (Table S1). There were no significant differences in thermal tolerance between Augusta Creek and Gull Creek, excluding Gull Lake. Gull Lake was significantly different in CT_max_ compared with upstream and downstream sites in Gull Creek and all sites in Augusta Creek. Interestingly, we did not find significant differences in CT_max_ between headwater and downstream locales in the same tributary, except for Gull Lake (Figure S4). We found a significant, positive correlation between water temperature and CT_max_ (Figure 5a; *r*_s_ = 0.3664, *p* < .001) and between average maximum air temperature and CT_max_ (Figure S5; *r*_s_ = 0.4397, *p* < .001). Cooler tributaries such as Augusta Creek displayed lower CT_max_, while warmer tributaries such as Wabascon Creek had higher CT_max_. We also found a significant, positive correlation between water temperature and average maximum air temperature for each site (Figure S6; *r*_s_ = 0.4075, *p* < .001). Although we did not detect any variance in our model associated with year as a random effect, we resampled a subset of 2017 sites to determine whether yearly variation in CT_max_ existed. KFB was the only site to display significant differences in CT_max_ between years (*t*(21) = −3.4052, *p* = .003). Excluding resamples, a positive correlation still existed between site water temperature (*r*_s_ = 0.4297, *p* < .001) and CT_max_, and average maximum air temperature (*r*_s_ = 0.5684, *p* < .001) and CT_max_.

## 4 DISCUSSION

In the face of anthropogenic change and novel stress, populations may persist if they have high plasticity and/or the necessary genetic variation to facilitate evolutionary rescue (Gonzalez et al., 2013; Hoffmann & Sgrò, 2011). In this study, we showed a strong correlation between genome‐wide diversity (heterozygosity) and CT_max_, whereby sites with the highest observed heterozygosity also tended to have the highest CT_max_. We also found support for putative adaptive differentiation in the sense that CT_max_ and morphology tended to diverge as expected based on variation in stream temperature and habitat, respectively. Overall, our findings of fine‐scale differentiation owing to both extrinsic (environmental conditions) and intrinsic (genetic diversity) factors point to the importance of characterizing intraspecific variation and suggest population‐level differences in vulnerability in a common, yet sensitive species.

Variation in biotic and abiotic conditions between lakes and streams often generates intraspecific phenotypic divergence. Our results, indicating that Gull Lake (GLD) individuals have reduced body depth and superior mouth positions compared to stream darters, are consistent with other examples of intraspecific ecotypic divergences. For example, threespine stickleback (*Gasterosteus aculeatus*) occupying both lakes and streams show marked differences in body size, life‐history traits, and other morphological characteristics (Hendry et al., 2002; Moore & Hendry, 2005; Moser et al., 2012). Individuals from lake populations display slender or streamlined bodies, with relatively shallower caudal peduncle depth, compared to their stream counterparts (Moore & Hendry, 2005; Ravinet et al., 2013; Sharpe et al., 2008). Although streamlined body shapes are often adaptive in high flow stream environments due to reduced drag (Cureton & Broughton, 2014; Franssen et al., 2013; Langerhans, 2008), other studies, including a study of different lake‐occupying *Etheostoma* species (Krabbenhoft et al., 2009), have found more streamlined body shapes in lake populations (McGuigan et al., 2003; Meyers & Belk, 2014). Our results match these latter studies, with more streamlined body shapes in Gull Lake individuals. This highlights how selective pressures other than flow, such as predation, behavior, or foraging ecology, may drive the morphological differences we observed (Collin & Fumagalli, 2011; Landy & Travis, 2015; Saint‐Laurent et al., 2003). The Gull Lake site in our study, and Lake Phalen in Minnesota (Katula, 2013), represent unique opportunities to study the generality of lake versus stream adaptation in rainbow darters. Making use of these lake occurrences of rainbow darters in future studies characterizing additional adaptive phenotypes and outlier loci would be of interest.

Our finding of environment‐associated differentiation in CT_max_ could be due to adaptive evolution or variation in phenotypic plasticity. Heterogeneous local conditions within and across tributaries potentially drove this fine‐scale phenotypic divergence in CT_max_ to optimally match individuals to their environmental thermal regimes. The positive correlations we documented between water temperature, genetic diversity, and CT_max_ provide some evidence for adaptive differentiation associated with environmental differences. The observed heterogeneity in local thermal conditions may drive this differentiation on the molecular level. Specifically, CT_max_ variability is dependent on the upregulation of heat shock proteins (HSP) that occurs when individuals are faced with acute high temperature stress (Basu et al., 2002; Feder & Hofmann, 1999; Iwama et al., 1998). Intraspecific variability in expression of HSP and subsequently CT_max_ may correspond to locally divergent ecological conditions (Narum & Campbell, 2015; Oksala et al., 2014). Our finding of environmentally associated differentiation for CT_max_ across sites elude to the possibility that HSP expression may be selected according to fine‐scale variation in local thermal regimes. On the other hand, site variation in CT_max_ can also reflect phenotypic plasticity in expression of HSP across different thermal regimes (Healy & Schulte, 2012; Schaefer & Ryan, 2006). Although we found a positive trend between site CT_max_ and water temperature, we were unable to obtain water temperature averaged over multiple time points and caution against over‐interpretation of this result. However, we did find a significant positive trend between our single point estimates of water temperature and maximum air temperature at each site, averaged throughout the summer. This suggests our single point estimates do reflect biologically meaningful differences in water temperature across sites, although longer‐term, in‐situ water temperature monitoring would be ideal. Whether rainbow darter populations are locally adapted versus able to acclimate to different temperatures through phenotypic plasticity is an exciting future question.

We also found evidence for a positive effect of genetic variation on CT_max_, supporting the classic heterozygosity–fitness correlation (Frankham, 1996; Miller et al., 2014; Reed & Frankham, 2003). Similarly, our results are consistent with theory positing that populations with lower genetic diversity have reduced adaptive potential or decreased stress tolerance (Frankham, 2005). These heterozygosity–fitness correlations (HFC) have generally relied on microsatellite markers and found weak signals, but genome‐wide estimates of heterozygosity using SNPs can be strong predictors for individual fitness across phenotypes in natural populations (Lemopoulos et al., 2019; Hoffman et al., 2014; Miller et al., 2014; but see Yates et al., 2019). Specifically, our finding that sites with increased genetic diversity have a higher CT_max_ suggests that individuals with higher genetic variation may be better at withstanding abrupt increases in water temperatures in the wild. Similar relationships have been documented in several other fish species, suggesting some generality in this pattern. In eastern mosquitofish (*Gambusia holbrooki*), a population found in a nuclear reactor effluent pond with near‐lethal temperatures had higher heterozygosity than surrounding natural populations (Meffe et al., 1995). Populations of Trinidadian guppies (*Poecilia reticulata*) with recent histories of gene flow had higher CT_max_ compared with neighboring populations with strong genetic drift and no gene flow (Fitzpatrick & Reid, 2019). Finally, in the least killifish (*Heterandria formosa*), experimentally bottlenecked populations having undergone declines in genetic diversity were less likely to evolve an adaptive response (increased CT_max_) when compared to nonbottlenecked populations (Klerks et al., 2019). Our study highlights the potential role of intraspecific genetic diversity in shaping variation in CT_max_. In particular, isolated populations with increased relatedness and reduced adaptive potential owing to increased genetic drift and inbreeding may be especially vulnerable to thermal stress (Bijlsma and Loeschcke 2012). Given recent work illustrating CT_max_ and thermal adaptation as a highly polygenic trait, our finding of a correlation between heterozygosity and CT_max_ underscores the importance of considering genome‐wide diversity for understanding stress tolerance (Bay & Palumbi, 2014; Dayan et al., 2019; Rose et al., 2018).

When comparing models that incorporated effects of both local thermal regime and heterozygosity on CT_max_, we were unable to distinguish between only site or the additive effects of site and heterozygosity as the best‐supported model. Thus, a combination of environmental (extrinsic) and genetic (intrinsic) factors likely explains the described variation in CT_max_ with some degree of phenotypic expression likely due to plasticity. We found evidence for plastic responses in one site (Kellogg Forest, KFB), where individuals sampled in 2018 had significantly higher CT_max_ than the previous year. Conversely, UAC, in the same tributary, did not display a plastic CT_max_ response across years despite temperature variability. This site also had the lowest genetic diversity and highest relatedness, suggesting a possible constraint on plastic responses. Although no study has directly tested the interplay between CT_max_, genetic diversity, and plasticity in darters, seasonal variation in CT_max_ has been shown in darters across temporal ranges (Hlohowskyj & Wissing, 1985). Interestingly, despite phenotypic plasticity being traditionally considered inversely related to heterozygosity (Pigliucci, 2005; Scheiner, 1993), there is some evidence in *Drosophilia* that heterozygous individuals have higher plasticity (Rocha & Klaczko, 2016). In our system, it is likely that variation in CT_max_ is to some extent plastic within a range of temperatures to which individuals are locally conditioned, but the adaptive potential for increased CT_max_ could be constrained by a lack of genetic diversity (such as UAC displaying nonsignificant temporal variation). Importantly, plastic mechanisms can also promote evolutionary change as well, through the accommodation or assimilation of traits in response to selection pressures (Kelly, 2019; Pigliucci et al., 2006, Pigliucci 2005). In order to disentangle the difference between plasticity and local adaptation, future studies are needed focusing on rearing rainbow darters from different thermal regimes for multiple generations in a common garden, as well as identifying loci associated with variation in CT_max_.

Contemporary population structure can impact the spread of adaptive genetic variation through evolutionary processes such as gene flow or genetic drift (Lande, 1976; Slatkin, 1987; Tigano & Friesen, 2016; Willi et al., 2006). Despite the fine‐scale scope of our study, we did find some evidence of population structure, which contrasts to similar darter studies that found no discernable genetic structure (Davis et al., 2015; Washburn et al., 2020). The two genetic clusters identified did not correspond to geographic stream distance. We suggest three possible explanations for the observed patterns. Altered stream connectivity through culverts or dams between sample sites may have led to reduced gene flow between proximate sites on a contemporary timescale. Alternatively, observed population genetic structure could be due to selection against migrants due to isolation by environment (Sexton et al., 2014; Wang & Bradburd, 2014). Given that a previous phylogenetic study indicated that rainbow darter populations in the Great Lakes basin originated from a common glacial refugium (Ray et al., 2006), and the lack of isolation by distance found in other drainages (Haponski et al., 2009), a final explanation is that restricted migration and genetic drift during postglacial colonization are potential forces shaping structure at the scale explored here.

Taken together, results from our study highlight the importance of understanding how ecological and evolutionary processes interact to affect intraspecific variation, adaptive potential, and vulnerability to environmental stress. Most darter species, including rainbow darters, are comprised of locally patchy populations with limited dispersal (Craig, 2008; Hicks & Servos, 2017). Fine‐scale population structure has been documented in several darter species (Blanton et al., 2019; Camak & Piller, 2018; Fitzpatrick et al., 2014) including isolation by distance in rainbow darters (Davis et al., 2015). Limited connectivity and “small population problems” associated with genetic drift likely contribute to the threatened conservation status of many darter species. However, the extent of local phenotypic variation and the effects of genetic variation on adaptive potential and stress tolerance have generally not been explored in this hyper‐diverse group of fishes. Our study points to interesting potential interactions among gene flow, selection, and the environment occurring on a highly spatial scale. For example, we found morphological differentiation between lake and stream habitats and environment‐associated patterns of CT_max_ suggestive of adaptive differentiation. At the same time, sites with lower genetic variation and increased relatedness appear to be more vulnerable to abrupt stress, suggesting that maintaining high genetic variation, possibly through gene flow, could be an important buffer against environmental change. Therefore, variation in dispersal capacity and gene flow could impact the spread of adaptive phenotypic and genetic variation that is critical for buffering against stress and enabling population persistence in this system.

Our study adds to the body of literature highlighting the link between genome‐wide diversity and stress tolerance. Furthermore, this is to our knowledge the only study in a darter species to specifically link individual genome‐wide diversity to CT_max_. Broadly, adaptive potential is consequential for populations to withstand novel stressors resulting from anthropogenic defaunation (Frankham, 2005; Hansen et al., 2012; Hoffmann & Sgrò, 2011). Characterizing intraspecific adaptive potential and stress tolerance is a critical step to identifying at‐risk populations before their decline. In the context of the ongoing defaunation period, the concern is shifting toward conservation efforts that include populations of widespread and common species (Gaston & Fuller, 2008; Hobbs & Mooney, 1998; McCallum, 2015). Local extinctions of common species are rapidly occurring or predicted to happen during the current period of “biological annihilation,” but remain largely undocumented until urgent conservation action is needed (Ceballos et al., 2017). Given the expected water temperature increases in the Mississippi and Great Lake drainages of ~2°C by the end of the century (van Vliet et al., 2012, 2013), our study illustrates a species of cool‐water fish that is potentially sensitive to future warming events and environmental change, and highlights the variability in population extinction risk. Future studies will require an integration of functional genetic diversity to phenotypic correlations, genome‐wide scans for adaptive outlier loci, transcriptomics, and common garden assays to determine the adaptive potential of populations across a species range. Combining this knowledge with contemporary population structure can yield a powerful framework for forecasting how populations will persist under increasingly stressful conditions.

## Supporting information

Supplementary Material

## Data Availability

Data for this study are available at https://doi.org/10.5061/dryad.4b8gtht9v. Sequence data are available at NCBI SRA under BioProject number PRJNA661476.
