# Supplementary material for: Genome‐wide diversity and habitat underlie fine‐scale phenotypic differentiation in the rainbow darter (Etheostoma caeruleum)
Source: Evol Appl. 2020 Oct 7;14(2):498–512. doi: 10.1111/eva.13135 (PMC7896715; doi:10.1111/eva.13135)
Supplement: Supplementary file 1 — Supplementary Material [file EVA-14-498-s001.docx]

**Table S1.** Pairwise site differences for body shape (below) and critical thermal maximum (CT_max_, above). Significant differences are indicated in bold (p<0.05). Values for CT_max_ are adjusted using the false discovery rate (FDR), while for body shape 10,000 permutations were run.


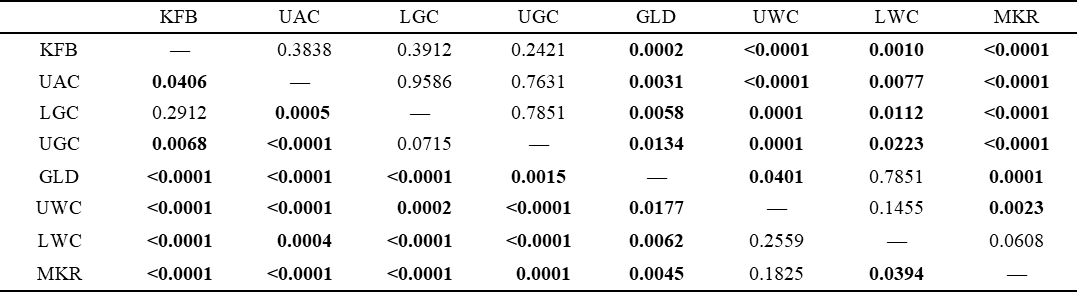


**Table S2.** Summary of the number of reads retained following each filtering step across both libraries.

**
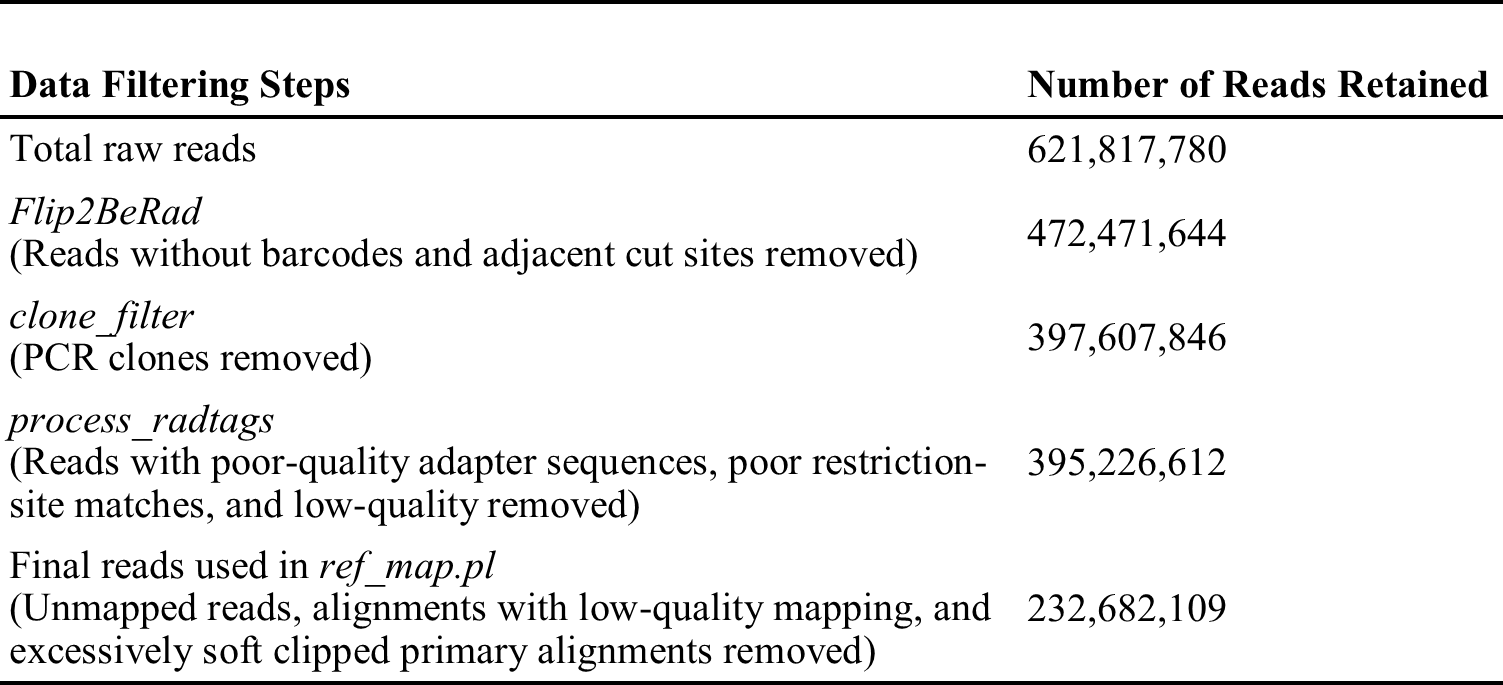
**

**Table S3.** Reads retained from the *process_radtags* function and the mean coverage metrics per individual. Summary statistics are given for both the entire dataset with all individuals (N=127) and the final filtered dataset (N=119, individuals filtered using *VCFtools* denoted by an asterisk).

| **Sample ID** | **Total Reads Prior *process_radtags*** | **Retained Reads After *process_radtags*** | **Proportion Retained Reads** | **Number of SNP loci** | | **Mean Depth** |
| --- | --- | --- | --- | --- | --- | --- |
| KFB1 | 4896936 | 4887774 | 0.998129 | 7818 | 37.3929 | |
| KFB3 | 2691938 | 2678965 | 0.995181 | 7785 | 20.6148 | |
| KFB4 | 2274960 | 2257908 | 0.992504 | 7750 | 17.3519 | |
| KFB5 | 2321636 | 2296920 | 0.989354 | 7737 | 17.606 | |
| KFB6 | 3213800 | 3185185 | 0.991096 | 7807 | 24.8144 | |
| KFB7 | 1630226 | 1620901 | 0.99428 | 7530 | 12.6653 | |
| KFB8 | 1503464 | 1487531 | 0.989402 | 7429 | 11.3796 | |
| KFB9 | 1938044 | 1919729 | 0.99055 | 7662 | 15.4105 | |
| KFB10 | 2751490 | 2729980 | 0.992182 | 7791 | 20.9435 | |
| KFB11 | 3382320 | 3351293 | 0.990827 | 7802 | 25.0274 | |
| KFB12 | 5631690 | 5620902 | 0.998084 | 7771 | 42.9749 | |
| KFB13 | 3351844 | 3344189 | 0.997716 | 7798 | 24.8951 | |
| KFB14 | 1454460 | 1413544 | 0.971869 | 7337 | 9.82554 | |
| KFB15 | 1680840 | 1661215 | 0.988324 | 7617 | 12.6379 | |
| KFB16* | 8636 | 8266 | 0.957156 | 85 | 1.28235 | |
| UAC1 | 1421558 | 1413024 | 0.993997 | 7270 | 10.8224 | |
| UAC2 | 1943758 | 1927912 | 0.991848 | 7703 | 14.6246 | |
| UAC3 | 5431110 | 5419909 | 0.997938 | 7840 | 40.6972 | |
| UAC4 | 2646332 | 2631061 | 0.994229 | 7802 | 19.868 | |
| UAC5 | 1615886 | 1598784 | 0.989416 | 7572 | 11.9595 | |
| UAC6 | 2049404 | 2032005 | 0.99151 | 7739 | 15.069 | |
| UAC7 | 1670850 | 1666661 | 0.997493 | 7650 | 12.8795 | |
| UAC8 | 1991868 | 1985273 | 0.996689 | 7744 | 15.1736 | |
| UAC9* | 62174 | 45530 | 0.7323 | 82 | 1.46341 | |
| UAC10 | 3451706 | 3442110 | 0.99722 | 7845 | 26.4161 | |
| UAC11 | 4123636 | 4107513 | 0.99609 | 7829 | 31.9283 | |
| UAC12 | 479560 | 477615 | 0.995944 | 5188 | 4.75482 | |
| UAC13 | 5178852 | 5165973 | 0.997513 | 7832 | 40.1387 | |
| UAC14 | 3335562 | 3322695 | 0.996142 | 7818 | 25.0421 | |
| UAC15 | 2374588 | 2356129 | 0.992226 | 7780 | 18.1067 | |
| LGC1 | 1121664 | 1117164 | 0.995988 | 6581 | 8.99742 | |
| LGC2 | 1684740 | 1674387 | 0.993855 | 7458 | 12.6763 | |
| LGC3 | 3188030 | 3179474 | 0.997316 | 7810 | 24.5375 | |
| LGC4 | 4376358 | 4353890 | 0.994866 | 7827 | 32.6741 | |
| LGC5 | 3119152 | 3110488 | 0.997222 | 7808 | 23.8281 | |
| LGC6 | 4697936 | 4672734 | 0.994636 | 7816 | 36.7743 | |
| LGC7 | 3210948 | 3202023 | 0.99722 | 7803 | 25.4929 | |
| LGC8 | 1891300 | 1883694 | 0.995978 | 7668 | 14.8912 | |
| LGC9 | 2805138 | 2798188 | 0.997522 | 7786 | 22.0213 | |
| LGC10 | 4106814 | 4090575 | 0.996046 | 7819 | 31.0029 | |
| LGC11 | 6000460 | 5979225 | 0.996461 | 7789 | 46.9901 | |
| LGC12 | 3262488 | 3252586 | 0.996965 | 7815 | 25.6897 | |
| LGC13 | 3650828 | 3641775 | 0.99752 | 7805 | 27.9221 | |
| LGC14 | 4074194 | 4062938 | 0.997237 | 7807 | 31.1359 | |
| LGC15 | 1937144 | 1931157 | 0.996909 | 7679 | 14.9284 | |
| UGC1 | 7662536 | 7645221 | 0.99774 | 7764 | 60.3841 | |
| UGC2 | 2408202 | 2400496 | 0.9968 | 7756 | 18.7842 | |
| UGC3 | 3109090 | 3099528 | 0.996925 | 7804 | 24.0037 | |
| UGC4 | 3436126 | 3421206 | 0.995658 | 7826 | 27.093 | |
| UGC5 | 2885616 | 2876923 | 0.996987 | 7810 | 22.9635 | |
| UGC6 | 8664598 | 8645885 | 0.99784 | 7742 | 67.8835 | |
| UGC7 | 4861288 | 4845624 | 0.996778 | 7787 | 37.3329 | |
| UGC8 | 2393420 | 2387817 | 0.997659 | 7723 | 19.432 | |
| UGC9 | 1523502 | 1519721 | 0.997518 | 7502 | 12.183 | |
| UGC10 | 2172946 | 2167195 | 0.997353 | 7682 | 16.98 | |
| UGC11 | 4200848 | 4190956 | 0.997645 | 7806 | 31.9965 | |
| UGC12 | 2476096 | 2469195 | 0.997213 | 7764 | 18.9745 | |
| UGC13 | 2020492 | 2015469 | 0.997514 | 7685 | 15.0069 | |
| UGC14 | 1571792 | 1565979 | 0.996302 | 7446 | 12.1097 | |
| UGC15 | 3591586 | 3583475 | 0.997742 | 7818 | 26.8949 | |
| GDL1 | 3476722 | 3468622 | 0.99767 | 7815 | 26.8212 | |
| GLD2 | 6816298 | 6796606 | 0.997111 | 7781 | 54.2268 | |
| GLD3 | 2162788 | 2157789 | 0.997689 | 7690 | 16.6036 | |
| GLD4 | 5061974 | 5050209 | 0.997676 | 7820 | 40.1098 | |
| GLD5* | 39104 | 37327 | 0.954557 | 167 | 1.60479 | |
| GLD6 | 3994854 | 3985646 | 0.997695 | 7833 | 29.5507 | |
| GLD7 | 1916774 | 1911348 | 0.997169 | 7690 | 15.1077 | |
| GLD8 | 2342420 | 2335461 | 0.997029 | 7705 | 18.4767 | |
| GLD9 | 1838382 | 1832851 | 0.996991 | 7663 | 14.5913 | |
| GLD10* | 18720 | 16022 | 0.855876 | 90 | 1.46667 | |
| GLD11* | 30508 | 27933 | 0.915596 | 172 | 1.55233 | |
| GLD12 | 3475292 | 3467240 | 0.997683 | 7809 | 27.0675 | |
| GLD13 | 3944992 | 3936709 | 0.9979 | 7805 | 31.011 | |
| GLD14 | 3981066 | 3970201 | 0.997271 | 7828 | 31.1689 | |
| GLD15 | 5591596 | 5576549 | 0.997309 | 7822 | 43.6652 | |
| GLD17 | 4012606 | 3977458 | 0.991241 | 7815 | 31.0289 | |
| GLD18 | 2139696 | 2132314 | 0.99655 | 7691 | 16.3578 | |
| GLD19 | 3628810 | 3599161 | 0.99183 | 7784 | 27.162 | |
| GLD20* | 149628 | 149352 | 0.998155 | 1950 | 2.75026 | |
| GLD21 | 3218856 | 3204592 | 0.995569 | 7800 | 24.6503 | |
| GLD22 | 1509730 | 1505150 | 0.996966 | 7539 | 12.4745 | |
| GLD23 | 5411656 | 5400585 | 0.997954 | 7797 | 40.3519 | |
| GLD24 | 3273488 | 3265436 | 0.99754 | 7826 | 25.094 | |
| GLD25 | 4662020 | 4650573 | 0.997545 | 7829 | 35.4748 | |
| UWC1 | 4275162 | 4264611 | 0.997532 | 7810 | 34.0328 | |
| UWC2 | 1431090 | 1423718 | 0.994849 | 7007 | 11.7357 | |
| UWC3 | 5870244 | 5853841 | 0.997206 | 7781 | 47.4728 | |
| UWC4 | 3403286 | 3393811 | 0.997216 | 7799 | 25.8968 | |
| UWC5 | 4576374 | 4565407 | 0.997604 | 7816 | 35.3968 | |
| UWC6 | 4009218 | 3999404 | 0.997552 | 7810 | 32.6421 | |
| UWC7 | 3381060 | 3373665 | 0.997813 | 7811 | 25.9009 | |
| UWC8 | 1514740 | 1510418 | 0.997147 | 7484 | 11.932 | |
| UWC9 | 3442772 | 3429188 | 0.996054 | 7808 | 27.0505 | |
| UWC10 | 5313640 | 5301316 | 0.997681 | 7790 | 40.6074 | |
| UWC11 | 1454116 | 1448486 | 0.996128 | 7300 | 11.2451 | |
| UWC12 | 1493312 | 1487454 | 0.996077 | 7446 | 11.7901 | |
| UWC13 | 5983562 | 5968259 | 0.997442 | 7752 | 40.7798 | |
| UWC14 | 8969432 | 8947756 | 0.997583 | 7706 | 63.2427 | |
| UWC15 | 2119642 | 2099351 | 0.990427 | 7647 | 15.1665 | |
| LWC1 | 3179480 | 3164287 | 0.995222 | 7793 | 23.0344 | |
| LWC2 | 3574750 | 3559148 | 0.995635 | 7782 | 25.9528 | |
| LWC3 | 1798960 | 1793877 | 0.997174 | 7566 | 13.6844 | |
| LWC4 | 2525278 | 2515468 | 0.996115 | 7702 | 18.9138 | |
| LWC5 | 1884198 | 1865303 | 0.989972 | 7401 | 13.1158 | |
| LWC6 | 2918620 | 2894792 | 0.991836 | 7742 | 21.7384 | |
| LWC7 | 3330074 | 3318832 | 0.996624 | 7781 | 23.9703 | |
| LWC8 | 2998666 | 2990369 | 0.997233 | 7778 | 21.9955 | |
| LWC9 | 3518676 | 3484345 | 0.990243 | 7730 | 24.9766 | |
| LWC10 | 2720976 | 2691666 | 0.989228 | 7739 | 19.5255 | |
| LWC11 | 2012610 | 1996886 | 0.992187 | 7581 | 14.3295 | |
| LWC12* | 30930 | 25661 | 0.829648 | 118 | 1.38983 | |
| MKR1 | 1867678 | 1861817 | 0.996862 | 7170 | 13.9753 | |
| MKR2 | 1684088 | 1677518 | 0.996099 | 7477 | 13.2521 | |
| MKR3 | 1979972 | 1964684 | 0.992279 | 7597 | 15.0021 | |
| MKR4 | 1973626 | 1961661 | 0.993938 | 7612 | 14.5 | |
| MKR5 | 3941380 | 3930784 | 0.997312 | 7780 | 28.7992 | |
| MKR6 | 2114792 | 2103244 | 0.994539 | 7666 | 15.7387 | |
| MKR7 | 4876528 | 4864029 | 0.997437 | 7784 | 35.1888 | |
| MKR8 | 5268186 | 5254018 | 0.997311 | 7771 | 37.9079 | |
| MKR9* | 32808 | 27443 | 0.836473 | 177 | 1.42373 | |
| MKR10 | 5199136 | 5181371 | 0.996583 | 7786 | 38.9468 | |
| MKR11 | 5667104 | 5649730 | 0.996934 | 7805 | 40.0425 | |
| MKR12 | 2023888 | 2017805 | 0.996994 | 7636 | 15.3096 | |
| MKR13 | 8142880 | 8103703 | 0.995189 | 7745 | 56.7717 | |
| MKR14 | 7363198 | 7335678 | 0.996262 | 7761 | 52.7445 | |
| MKR15 | 3541696 | 3529543 | 0.996569 | 7745 | 25.8026 | |
| MKR16 | 2108040 | 2099647 | 0.996019 | 6776 | 15.465 | |
| **Total** | **396828308** | **395226612** |  | **916088** |  | |
| Min (All)  Min (Filtered) | 8636  479560 | 8266  477615 | 0.7323  0.9719 | 82  5188 | 1.282  4.755 | |
| Max (All)  Max (Filtered) | 8969432  8969432 | 8947756  8947756 | 0.9981  0.9981 | 7845  7845 | 67.883  67.883 | |
| **Mean (All)**  **Mean (Filtered)** | **3124632.346**  **3331561.435** | **3112020.567**  **3318395.613** | **0.9886**  **0.9955** | **7213.291**  **7674.345** | **23.796**  **25.287** | |
| SD (All)  SD (Filtered) | 1790427.877  1655674.832 | 1786980.119  1652794.198 | 0.033935744  0.003297128 | 1808.625  303.243 | 13.294  12.381 | |

**Table S4.** Pairwise genetic distance (F_ST_, bottom) and geographic distance (km, top) between the eight sample sites. Geographic distance was measured following stream networks.


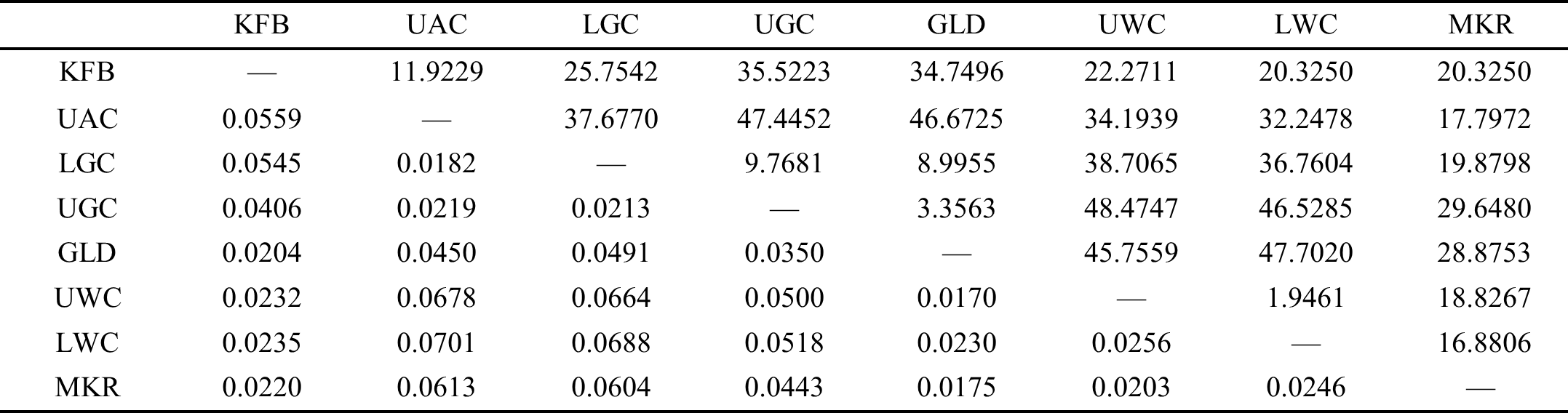


**Table S5.** Model selection using AIC. The response variable (CT_max_) was explored through different linear mixed-effects models containing different fixed effects, including sample site (*E*), observed heterozygosity (*G*), the interaction between site and heterozygosity (*E x G*), sex, (*S*), and weight (*W*).


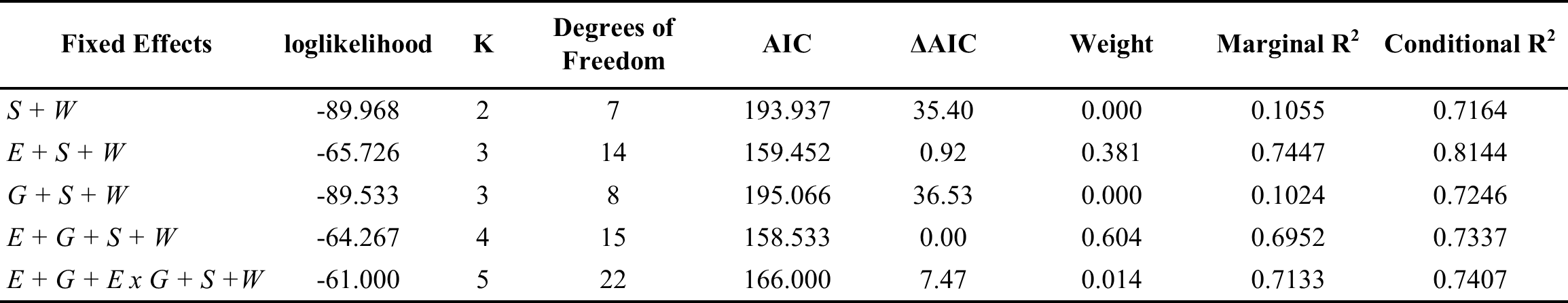


**Note:** K is the number of parameters in the model. ΔAIC is the difference in AIC between the best fitting model and the given model. Weight is the Akaike weight of the given model. Marginal R^2^ represents the portion of variance explained by the model for fixed effects only, while conditional R^2^ represents the portion of variance explained by the model including both fixed and random effects.

**Figures**

**Figure S1.** Morphospace produced by canonical variate analysis (CVA) conducted between the eight sites. Ellipses represent 95% confidence around the site mean for the two axes. Extremes for canonical variate one is represented by wireframe graphs below the x-axis. Extremes for canonical variate two is represented by wireframe graphs to the left of the y-axis.

**Figure S2.** Correlation testing for isolation by distance (IBD). Geographic distance (km) are distances between sample sites following stream network, while genetic distance is the F_ST_ calculated between sites using *stacks*.

**Figure S3.** Genetic distance between sites as explained by stream connectivity using *StreamTree*. The dotted line represents a one to one relationship between observed genetic distance (F_ST_ from *stacks*) and fitted distance (sum of the genetic distance through each stream section). Pairwise site comparisons in the blue section have higher than expected genetic distance, while those in the red section have lower than expected genetic distance.

**Figure S4.** Mean critical thermal maximum (CT_max_) per site. Error bars indicate 95% confidence intervals. Upper Augusta Creek (UAC) and Gull Lake (GLD) represent both 2017 and 2018 individuals, while for Kellogg Forest (KFB) both years are shown due to being significantly different (2018 represented by an asterisk). Letters represent distinct phenotypic groups adjusted using a false discovery rate (FDR).

**Figure S5.** Correlation between average June-August maximum air temperature and critical thermal maximum (CT_max_) at each site. Error bars indicate 95% confidence intervals with dashed error bars representing sites in which resampling was conducted in 2018.

**Figure S6.** Correlation between average June-August maximum air temperature and site water temperature. Open points represent sites in which resampling was conducted in 2018.

**Figure S1**

**

**

**Figure S2**

**
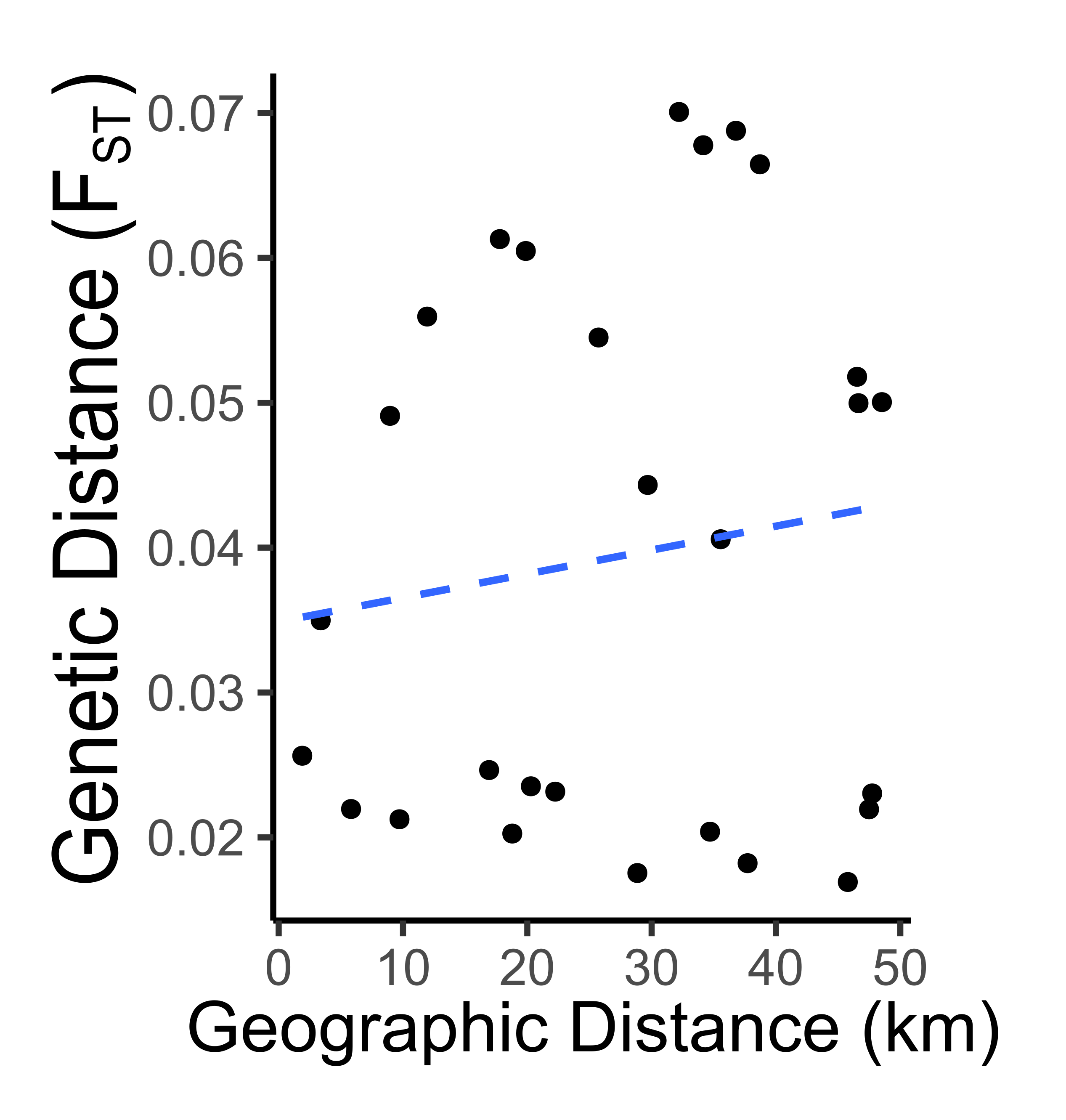
**

**Figure S3**

**
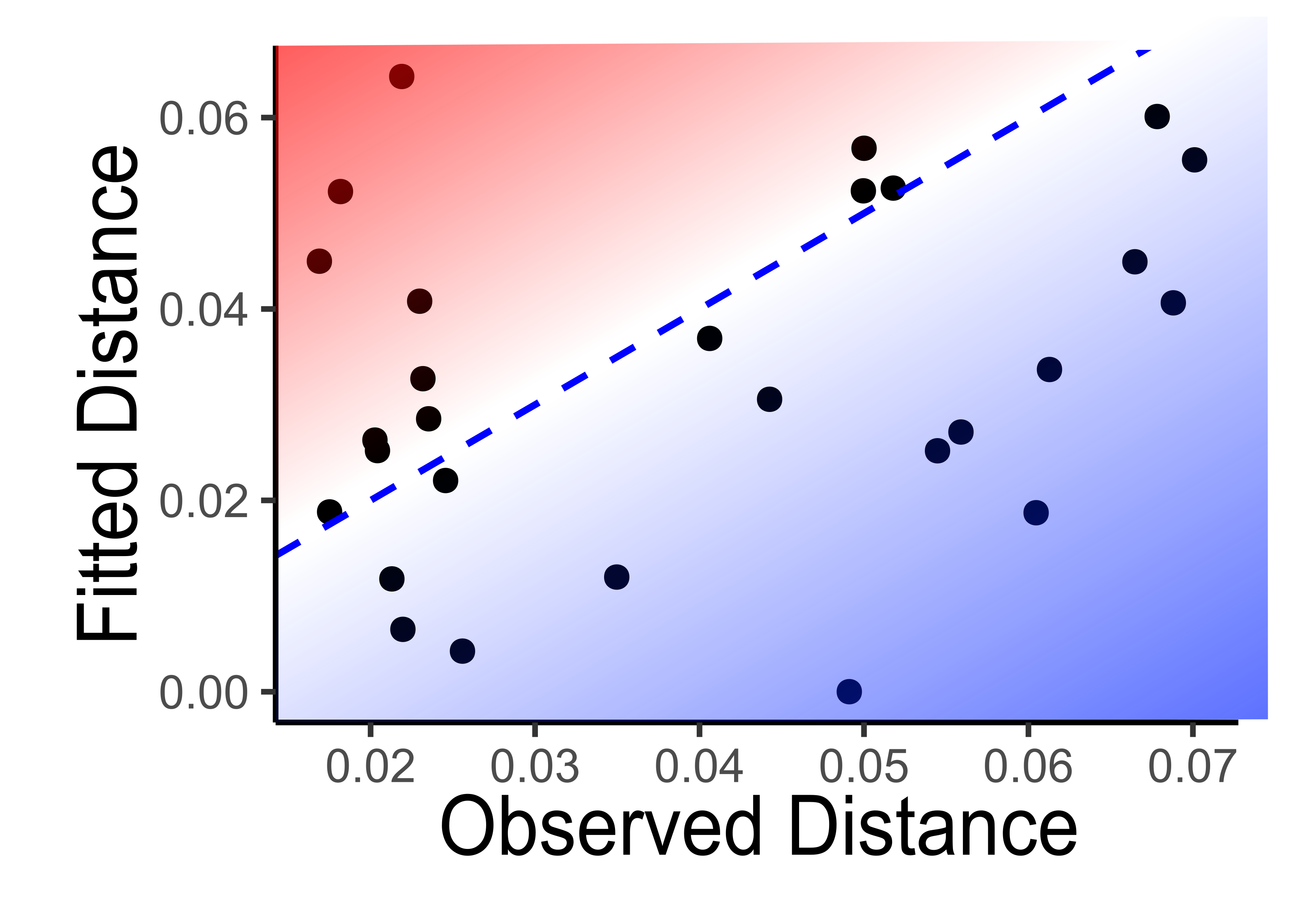
**

**Figure S4**

**
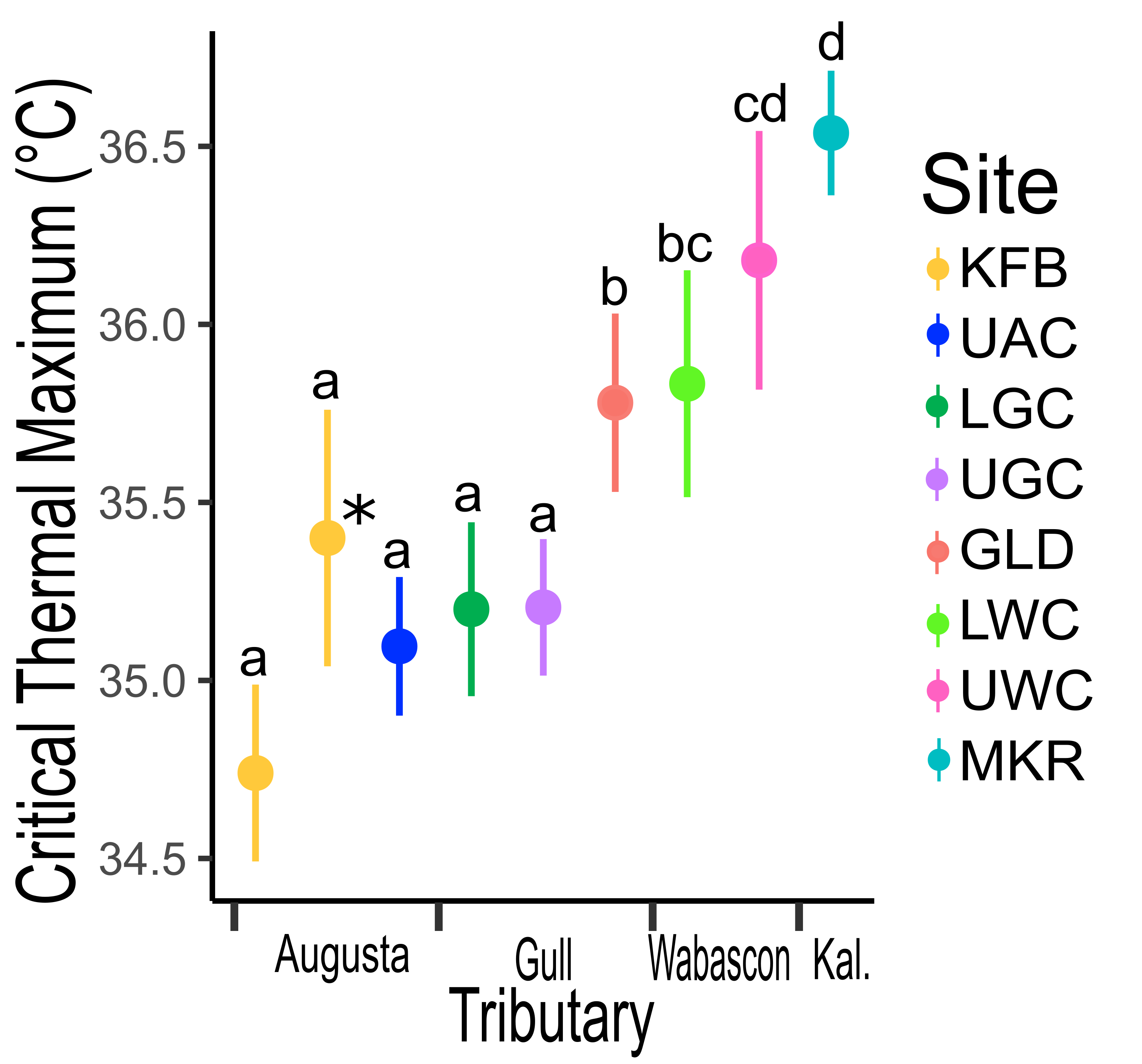
**

**Figure S5**

**
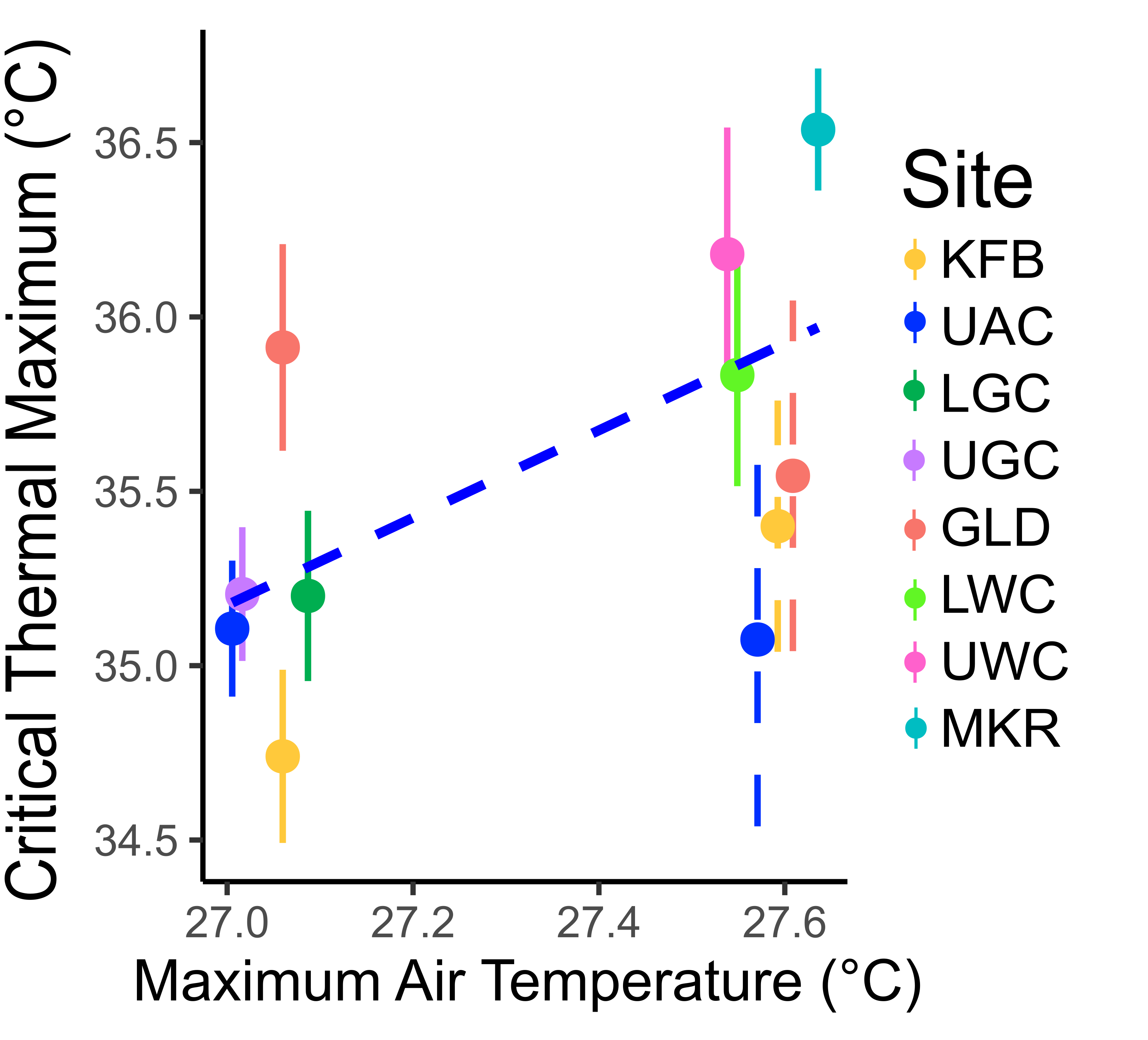
**

**Figure S6**

**
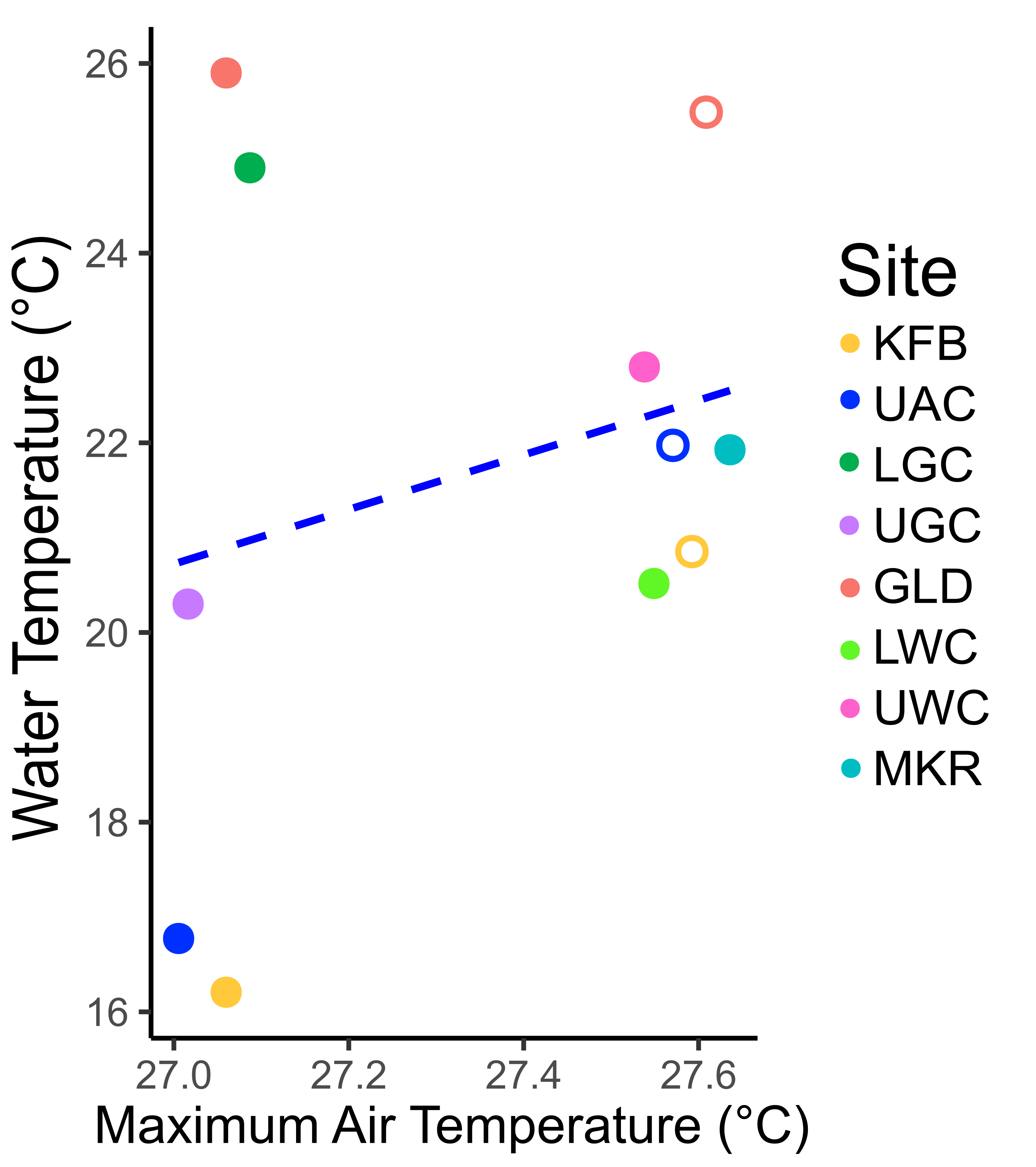
**

**
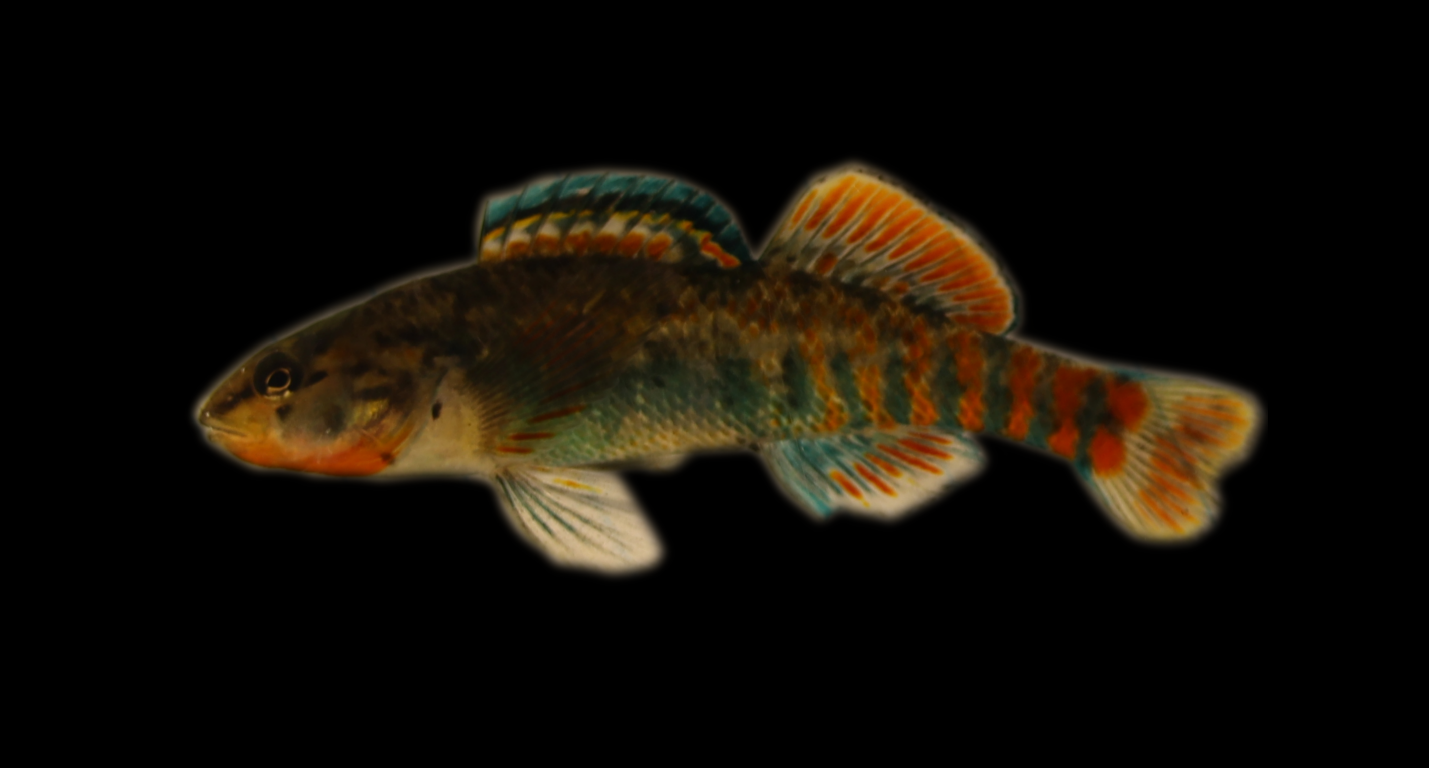
**
